# Increasing astrogenesis in the developing hippocampus induces autistic‐like behavior in mice via enhancing inhibitory synaptic transmission

**DOI:** 10.1002/glia.24091

**Published:** 2021-09-09

**Authors:** Juan Chen, Xiao‐Lin Ma, Hui Zhao, Xiao‐Yu Wang, Min‐Xin Xu, Hua Wang, Tian‐Qi Yang, Cheng Peng, Shuang‐Shuang Liu, Man Huang, Yu‐Dong Zhou, Yi Shen

**Affiliations:** ^1^ Department of Neurobiology and Department of General Intensive Care Unit of the Second Affiliated Hospital Zhejiang University School of Medicine Hangzhou China; ^2^ Department of Neurobiology and Department of Ophthalmology of the First Affiliated Hospital Zhejiang University School of Medicine Hangzhou China; ^3^ NHC and CAMS Key Laboratory of Medical Neurobiology, MOE Frontier Science Center for Brain Research and Brain‐Machine Integration, School of Brain Science and Brain Medicine Zhejiang University Hangzhou China; ^4^ Core Facilities Zhejiang University School of Medicine Hangzhou China; ^5^ Department of General Intensive Care Unit of the Second Affiliated Hospital Zhejiang University School of Medicine Hangzhou China; ^6^ Department of Pharmacology Zhejiang University City College School of Medicine Hangzhou China; ^7^ National Human Brain Bank for Health and Disease Hangzhou China

**Keywords:** astrogenesis, autistic‐like behavior, early development, excitatory‐inhibitory balance, GABAergic synaptic transmission, hippocampus

## Abstract

Autism spectrum disorder (ASD) is a heterogeneous neurodevelopmental disorder characterized primarily by impaired social communication and rigid, repetitive, and stereotyped behaviors. Many studies implicate abnormal synapse development and the resultant abnormalities in synaptic excitatory–inhibitory (*E*/*I*) balance may underlie many features of the disease, suggesting aberrant neuronal connections and networks are prone to occur in the developing autistic brain. Astrocytes are crucial for synaptic formation and function, and defects in astrocytic activation and function during a critical developmental period may also contribute to the pathogenesis of ASD. Here, we report that increasing hippocampal astrogenesis during development induces autistic‐like behavior in mice and a concurrent decreased *E*/*I* ratio in the hippocampus that results from enhanced GABAergic transmission in CA1 pyramidal neurons. Suppressing the aberrantly elevated GABAergic synaptic transmission in hippocampal CA1 area rescues autistic‐like behavior and restores the *E*/*I* balance. Thus, we provide direct evidence for a developmental role of astrocytes in driving the behavioral phenotypes of ASD, and our results support that targeting the altered GABAergic neurotransmission may represent a promising therapeutic strategy for ASD.

## Main Points

- Increasing hippocampal astrogenesis during development induces autistic‐like behavior and reduced *E*/*I* ratio via enhancing GABAergic inhibition.
- Suppressing the aberrantly enhanced GABAergic inhibition in CA1 rescues autistic‐like traits.

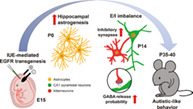

## 1 INTRODUCTION

Autism spectrum disorder (ASD) is a heterogeneous neurodevelopmental disorder characterized primarily by impaired social communication and rigid, repetitive, and stereotyped behaviors (Lord et al., 2018). Several ASD models postulate abnormal synapse development and the resultant abnormalities in synaptic excitatory–inhibitory (*E*/*I*) balance may underlie many features of the disease (Auerbach et al., 2011; Etherton et al., 2011; Nelson & Valakh, 2015; Sohal & Rubenstein, 2019), suggesting aberrant neuronal connections and networks are prone to occur in the developing autistic brain (Courchesne et al., 2019; Nelson & Valakh, 2015; Sakers & Eroglu, 2019). The hippocampus is one of the well‐documented brain regions implicated in ASD, since abnormal hippocampal circuits have been frequently observed in both ASD patients (Bauman & Kemper, 1985; Schumann et al., 2004; Sparks et al., 2002) and several ASD mouse models (e.g., *Shank1* ^−/−^ mice) (Hung et al., 2008; Sungur et al., 2017). Although neuron related molecular and circuit mechanisms have been extensively studied in ASD, glia dysfunction also contributes to ASD neuropathogenesis. In particular, astrocytes, the most abundant cell type in the mammalian brain, play an essential role in neural development by powerfully coordinating synapse formation and function, neuronal survival, and axon guidance (Allen & Eroglu, 2017; Eroglu & Barres, 2010). Structural and functional defects of astrocytes during a critical developmental period have been suggested in the pathogenesis of ASD (Sloan & Barres, 2014). Increased number of cytotoxic astrocytes, elevated GFAP expression, and abnormal expression patterns of AQP4 and CX43 have been observed in ASD patients (Ahlsén et al., 1993; DiStasio et al., 2019; Fatemi et al., 2008; Laurence & Fatemi, 2005) and astrocytic involvement in ASD is also supported by the experimental data obtained from ASD mouse models [e.g., *MeCP2* mutant mice and *Fmr1* knockout (KO) mice] (Ballas et al., 2009; Hodges et al., 2017; Lioy et al., 2011; Rakela et al., 2018). However, the role of astrocytes in ASD pathogenesis during brain development remains obscure.

Strong evidence shows that prenatal events, such as abnormal cell proliferation, might underlie early‐age brain growth defects that precede and produce postnatal phenotypic heterogeneity in ASD (Courchesne et al., 2007; Courchesne et al., 2019; Stoner et al., 2014). Astrocytes are differentiated from neural stem cells (NSCs) located in the ventricular and subventricular zones in the gliogenic phase of late gestation during brain development, in which the late embryonic and early postnatal periods are the most critical stages for astrogenesis (Namihira & Nakashima, 2013). Given the astrocyte's role in helping to shape neural circuits in the developing brain, this raises the question whether and how aberrant astrocyte development from the late prenatal to early postnatal stages, arguably the most important critical period in ASD (Courchesne et al., 2019), contributes to ASD pathogenesis. Here, we generated a mouse model of ASD with increased neonatal astrogenesis in the hippocampus and investigated the behavioral abnormalities as well as the excitatory and inhibitory synaptic transmission deficits in hippocampal CA1 area, to elucidate the critical role of astrocytes in ASD pathogenesis in the early developing brain.

## 2 MATERIALS AND METHODS

### 2.1 Animals

All procedures were carried out in accordance with the National Institutes of Health Guidelines for the Care and Use of Laboratory Animals and were approved by the Animal Advisory Committee at Zhejiang University. C57BL/6J (stock number 000664) mice were purchased from the Jackson Laboratory (Bar Harbor, ME). All mice were housed at the Animal Facility of Zhejiang University under a 12‐h light/dark cycle (lights on at 8:00) and constant temperature (22 ± 2°C) conditions with free access to food and water. Timed‐pregnant mice were used for in utero electroporation (IUE) and single‐housed after surgery and returned to the same cage rack. Mice were left undisturbed, except for weekly cage cleaning, until the pups were weaned at 3 weeks of age. Offspring were housed in same‐sex groups of two to five animals. For behavioral experiments, only male mice were used.

### 2.2 The *piggyBac* plasmids and IUE procedures

The *piggyBac* plasmids (including the helper plasmid: pGLAST‐PBase and the donor plasmids: pPBGFAP‐eGFP, pPBCAG‐epidermal growth factor receptor [EGFRs]) were kindly provided by Dr. Joseph LoTurco (Chen & LoTurco, 2012). To construct a donor plasmid pPBGFAP‐EGFR, the CAG sequence was replaced by GFAP sequence from pPBGFAP‐eGFP plasmid in the *KpnI‐HF* and *SpeI‐HF* sites by T4 DNA ligase. pPBGFAP plasmid was constructed by replacing CAG‐EGFR sequence with GFAP sequence in pPBCAG‐EGFR using *SpeI* and *NotI* sites. pGLAST‐PBase and pPBGFAP‐EGFR plasmids were used as experimental group (EGFR‐IUE mice), and pGLAST‐PBase and pPBGFAP plasmids were used as control groups (Ctrl‐IUE mice). In particularly, pPBGFAP‐eGFP plasmid was used specifically to examine the average transfection rates. IUE aiming hippocampal CA1 was performed in embryonic day 15 (E15) mice as previously described (Chen & LoTurco, 2012; Shen et al., 2016) with minor modifications. Briefly, E15 mice were anesthetized with a gas anesthetizer (MIDIMARK, Matrx VIP 3000; mix ratio 1.5, flow rate 1.0, consuming 1 ml isoflurane per 10 min) before surgery. To visualize the electroporating process, plasmids were mixed with 2 mg/ml Fast Green (Sigma‐Aldrich) which was used as a visual tracer. In all conditions, pPBGFAP (or pPBGFAP‐eGFP for test) and pPBGFAP‐EGFR were used at the final concentration of 1.5 and 4 μg/μl, respectively, while pGLAST‐PBase was used at the final concentration of 3 μg/μl. The smaller sweeps (0°–15°) were used to yield preferential transfection of CA1 subfield using the interaural line as a reference plane of 0° (Navarro‐Quiroga et al., 2007).

During surgery, the uterine horns were exposed and one lateral ventricle of each embryo was pressure injected with 1–1.5 μl of plasmid DNA. Injections were made by inserting a pulled glass microelectrode into the lateral ventricle through the uterine wall and embryonic membranes and injecting the content of the microelectrode by pressure pulses delivered with an electroporator (NEPA GENE, EDIT‐TYPE CUY‐21). Electronic pulses (40 V; 50 ms) were charged five times at intervals of 950 ms. Antibiotics were administered at a dose of 1 mg/kg s.c. for 1 day following surgery. To estimate the electroporation efficiency, coronal brain sections (30 μm) from E18 IUE embryos were cut using a freezing microtome (CM30503, Leica). Tissue sections were incubated with rabbit anti‐GFAP and Alexa Fluor 546 anti‐rabbit IgG , and then mounted onto glass slides with ProLong® Gold Antifade Reagent for fluorescent image (as mentioned in Section 2.3). For analysis of electroporated brains, at least three anatomically matched sections were quantified per brain from at least three embryos or pups from different mothers. The slices were imaged using a Nikon A1 laser‐scanning confocal microscope through a 20× objective (numerical aperture 0.75). Images were taken at a resolution of 1024 × 1024 pixels at room temperature (RT). The average IUE transfection rate (measured as the ratio of GFP and GFAP double‐positive cell number/GFAP‐positive cell number × 100%) in hippocampal CA1 from E18 tested mice were 20.1% ± 1.8% (ranging from the lowest rate of 11.7% to the highest rate of 29.1%; *n* = 14). Data from EGFR‐IUE mice with increased astrogenesis lower than 20% compared to Ctrl‐IUE mice were excluded.

### 2.3 Immunohistochemistry

Immunohistochemistry experiments were carried out as previously described (Shen et al., 2016). Mice were anesthetized with sodium pentobarbital (200 mg/kg, i.p.) and perfused transcardially with 4% paraformaldehyde (PFA) in PBS. Brains were extracted and post‐fixed in 4% PFA overnight and then moved to 30% sucrose in PBS. After brains were saturated (36 h), 30 μm‐thick coronal brain sections were cut using a freezing microtome (CM30503, Leica). Sections were processed as free‐floating sections. After being blocked in PBS containing 5% of normal donkey serum (NDS; Jackson Immuno Research 017‐000‐121), 1% bovine serum albumin (BSA; Sigma‐Aldrich), and 0.3% Triton X‐100 (Sigma‐Aldrich) for 1 h at RT, tissue sections were incubated with primary antibodies [rabbit anti‐GFAP (1:1000; Dako Z0334), mouse anti‐ALDH1L1 (1:1000; Sigma‐Aldrich MABN495), rabbit anti‐Ki67 (1:500; Abcam ab15580), mouse anti‐GAD67 (1:200; Sigma‐Aldrich MAB5406), rabbit anti‐CamKII (1:500; Cell Signaling Technology 4436), mouse anti‐VGLUT1 (1:100; Synaptic Systems 135511), rabbit anti‐VGAT (1:400; Synaptic Systems 131013), mouse anti‐gephyrin (1:500; Synaptic Systems 147021), mouse anti‐HuC/HuD (1:50; ThermoFisher Scientific A‐21271), rabbit anti‐Iba‐1 (1:500; Wako 019‐19741), rabbit anti‐EGFR (1:500; Proteintech 18986‐1‐AP)] overnight at 4°C in the blocking solution. Tissue sections were washed in PBS (15 min, three times), incubated with an appropriate secondary antibody [Alexa Fluor 546 anti‐mouse IgG (1:1000; ThermoFisher Scientific A10036), Alexa Fluor 546 anti‐rabbit IgG (1:1000; ThermoFisher Scientific A10040), Alexa Fluor 405 anti‐mouse IgG (1:500; ThermoFisher Scientific A‐31553 or Alexa Fluor 405 anti‐rabbit IgG (1:1000; Abcam ab175654)] for 1 h at RT. After being washed with PBS three times, sections were mounted onto glass slides with ProLong® Gold Antifade Reagent (ThermoFisher Scientific P36931) or 60% glycerol in PBS.

Images were captured using a Nikon A1 laser‐scanning confocal microscope through a 20× objective (numerical aperture 0.75) or 60× oil‐immersion objective (numerical aperture 1.40). All the images were taken at a resolution of 1024 × 1024 pixels at RT. Gain, threshold, and black levels were not subjected to change during individual experiments. For quantitative analysis, the stratum pyramidale (SP), stratum oriens (SO), stratum radiatum (SR), and stratum lacunosum‐moleculare (SLM) of CA1 and CA3 subfields of the hippocampus as well as the cortical layer VI area close to the hippocampus were imaged at 20× and magnified fivefold. The numbers of cells (astrocytes, neurons, and microglia) and immunosignal of proteins were estimated by calculating the normalized numbers of fluorescent cells (ALDH1L1, GFAP, GAD67, and Ki67) per unit area using NIH ImageJ software and/or the relative mean fluorescence intensities of the markers (ALDH1L1, GFAP, EGFR, and CaMKII) per unit area using MetaMorph (UIC), respectively. Synaptic puncta number and size were analyzed using Imaris and MetaMorph (UIC). The images were thresholded to outline immunopositive puncta, and the number and average size of puncta were detected and normalized with data from Ctrl‐IUE mice to determine synaptic density and strength. The numbers of astrocyte processes (branches) and process junctions (branching points) and the mean process length were analyzed using NIH ImageJ software to determine the morphology of GFAP‐positive astrocytes (Giocanti‐Auregan et al., 2016). All image analysis was done blind to the experimental condition. Data from EGFR‐IUE mice with increased astrogenesis lower than 20% compared to Ctrl‐IUE mice were excluded.

### 2.4 Behavioral tests

All behavioral tests were performed with age‐matched male littermates (5–6 weeks) during the day (light‐on period). Mice were handled for at least 2–3 min/day for 5 days until they were habituated to the operator. There were at least 1‐day rest periods between tests. All the apparatuses were cleaned with 75% ethanol and air‐dried between tests. All behavioral assays (including social interaction) were performed by examiner blinded to IUE groups. Data from EGFR‐IUE mice with increased astrogenesis lower than 20% compared to Ctrl‐IUE mice were excluded.

#### 2.4.1 Three‐chamber social interaction test

The three‐chamber social interaction test was performed to investigate sociability in mice, as described previously with minor modifications (Li et al., 2016; Ren et al., 2020). Mice (5–6 weeks) were tested in the apparatus consisted of a rectangular, three‐chambered box and a lid with a video camera (Logitech Carl Zeiss Tessar Webcam HD 1080P). Each chamber (20 cm × 40 cm × 20 cm) was divided by a clear plastic wall with a small square opening (5 cm × 8 cm). First, the mouse was placed in the apparatus and allowed to explore the environment freely for 5 min for habituation. Then, the mouse was gently guided to the center chamber, and its two entrances were blocked while a stranger mouse was placed in one side chamber. The position of stranger mouse was alternated between tests to prevent side preference. The two entrances were then opened to allow the subject mouse to explore the new environment freely for 10 min. All stranger mice were males of the same age and previously habituated to the plastic cage for 30 min during the previous day. The time spent in each chamber was automatically recorded and then calculated. We also calculated the difference index, which was the numerical difference between the times spent exploring the targets (Stranger versus Empty) divided by the total time spent exploring both targets. The data were analyzed using ANY‐maze Video Tracking System 4.98.

#### 2.4.2 Repetitive self‐grooming

Mice were scored for spontaneous grooming behaviors as previously described (Silverman et al., 2010). Each mouse was placed individually into a standard mouse cage (30 cm length × 25 cm wide × 20 cm high). A thin (1 cm) layer of bedding reduced neophobia, while preventing digging, a potentially competing behavior. A front mounted camera (Logitech Carl Zeiss Tessar Webcam HD 1080P) was placed approximately 50 cm from the cages to record the sessions. After a 10‐min habituation period in the test cage, each mouse was scored with a timer for 10 min for cumulative time spent grooming all body regions. A trained observer uninformed of the genotypes scored the videos.

#### 2.4.3 Open field test (OFT)

OFT was performed as previously described (Li et al., 2016). The open field apparatus was an open white plastic box (40 cm × 40 cm × 40 cm) evenly illuminated at 30 lux. Mice were placed in the center of the open field and allowed to explore for 300 s. The time spent in the central area (25 × 25 cm^2^) and the total locomotor distance were recorded with a video camera and analyzed using ANY‐maze Video Tracking System 4.98.

#### 2.4.4 Morris water maze (MWM) test

The tests were performed in a circular tank (120 cm in diameter and 60 cm in height) filled with opaque water at 22–24°C as previously described (He et al., 2019). The tank was divided into four quadrants with different navigation landmarks for each quadrant. Twenty‐four hours before the acquisition test, a visible platform task was performed by measuring the time spent to find a colorful flag placed on the top of a platform in a quadrant. The visible platform task was tested in each quadrant to avoid habituation. In the hidden platform acquisition test, mice were allowed to swim freely to search for the escape platform within 60 s. The platform location remained constant throughout the test. The time taken to reach the platform was recorded as the escape latency. The mouse was allowed to stay on the platform for 10 s after the hidden platform was found. If a mouse failed to find the platform within 60 s, the mouse was guided to the platform and stayed on the platform for 10 s, and the escape latency was recorded as 60 s for this trial. The same animal was then released from a new insertion point 4 min after the previous trial. The experiment was repeated four times per mouse each day for 5 days. The four animal insertion points were chosen to maintain a constant distance to the platform. The mean escape latency was calculated to evaluate the spatial learning ability. Twenty‐four hours after the hidden platform acquisition test, probe trials were conducted by removing the platform. Mice were placed in the diagonal quadrant of the hidden platform originally located and were allowed to swim freely in the pool for 60 s. The numbers of entries into the area where the original platform was located and crossings over the original platform were recorded. The data were analyzed by the WaterMaze 4.07 Software (ActiMetrics software).

### 2.5 Hematoxylin and eosin (H&E) staining

The brains were fixed in 4% PFA for 48 h and were dehydrated and paraffin embedded using the standard procedure. For H&E staining, after deparaffinization and rehydration, the slides (3 μm) were stained in hematoxylin solution for 8 min and then in eosin solution for 30 s to 1 min. Then slides were mounted with neutral balsam (Sinopharm Chemical Reagent). Images were obtained using a VS120 Olympus optical microscope through a 20× objective (numerical aperture 0.75).

### 2.6 Whole‐cell patch‐clamp recording

Mice (2‐ or 7‐week old) were anesthetized with sodium pentobarbital and the brain was then removed after decapitation. The brain areas containing hippocampus were dissected rapidly and transferred to a chamber filled with ice‐cold artificial cerebrospinal fluid (ACSF; in mM: 124 NaCl, 2 KCl, 1.25 KH_2_PO_4_, 2 MgSO_4_, 2 CaCl_2_, 26 NaHCO_3_, and 10 d‐Glucose, pH 7.4, 300 mOsm). Transverse hippocampal slices (300 μm) were cut with a tissue slicer (VT 1200S, Leica) and incubated in oxygenated (95% O_2_/5% CO_2_) ACSF. Slices were allowed to recover for ~30 min in ACSF at 32°C, and subsequently for 1 h at RT.

Whole‐cell recordings were performed as previously described (He et al., 2019; Smith et al., 2011; Zhou et al., 2009). Data from EGFR‐IUE mice with increased astrogenesis lower than 20% compared to Ctrl‐IUE mice were excluded. Hippocampal slices were then transferred to a recording chamber and perfused continuously with ACSF at 35°C bubbled with 95% O_2_/5% CO_2_ to ensure adequate oxygenation of slices. Whole‐cell recordings were performed on CA1 pyramidal neurons. Neurons were identified under infrared differential interference contrast (IR‐DIC) optics based on their location and morphology. Borosilicate glass (A‐M system) pipettes (3–5 MΩ) were pulled with a horizontal pipette puller (P97, Sutter instruments) and were filled with cesium‐based intracellular fluid (in mM: 100 CsCH_3_SO_3_, 20 KCl, 10 HEPES, 4 Mg‐ATP, 0.3 Tris‐GTP, 7 Tris_2_‐Phosphocreatine, 3 QX‐314; pH 7.3, 285–290 mOsm). Pipettes were connected to the headstage of a Heka EPC 10 amplifier (Heka Elektronik), and fast and slow capacitance as well as series resistance compensations were carefully adjusted. Liquid junction potentials were not corrected.

Spontaneous excitatory postsynaptic currents (sEPSCs) were recorded at a holding potential of −70 mV and spontaneous inhibitory postsynaptic currents (sIPSCs) were recorded at a holding potential of +10 mV in regular ACSF. *E*/*I* ratio was calculated as *E*/(*E* + *I*) in each pyramidal cell (Antoine et al., 2019). Spontaneous EPSCs and IPSCs under pharmacological isolation in CA1 pyramidal neurons were recorded as follows: sEPSCs were recorded at −70 mV in regular ACSF containing 10 μM bicuculline methiodide (BMI; Abcam) and the glass pipettes were filled with cesium‐based intracellular fluid; sIPSCs were recorded at −70 mV in regular ACSF containing 10 μM DNQX (Abcam) and 40 μM APV (Abcam) and the glass pipettes were filled with internal solution containing (in mM) 140 CsCl, 10 HEPES, 2 QX‐314, 3 Mg‐ATP, 0.1 EGTA, and 0.4 Na_3_GTP (pH 7.3, 285–290 mOsm). Miniature EPSCs (mEPSCs) were recorded at −70 mV in regular ACSF containing 0.5 μM tetrodotoxin (TTX; Abcam) and 10 μM BMI. mIPSCs were recorded at +10 mV in regular ACSF containing 0.5 μM TTX, 10 μM DNQX, and 50 μM APV. Series resistance was normally less than 20 MΩ and recordings exceeding 20% change in series resistance were terminated and discarded. Electrophysiological recordings were filtered at 2.0 kHz and digitized at 50 kHz. Individual events were counted and analyzed with MiniAnalysis (Synaptosoft). For kinetic analysis, only single‐event EPSCs/IPSCs with a stable baseline, sharp rising phase (10%–90% rise time), and exponential decay were chosen during visual inspection of the recording traces. Double‐ and multiple‐peak EPSCs/IPSCs were excluded. 300 s of recordings randomly chosen from five cells in each group were analyzed (Shen et al., 2016; Zhou et al., 2009).

Evoked EPSCs were elicited in the presence of 10 μM BMI using a bipolar stimulating electrode (CE2C75, FHC Inc.) placed in stratum radiatum 300 μm away from the recording site. The rectangle current pulses (duration: 200 μs, frequency: 20 Hz) were delivered via a constant‐current stimulator (SIU91A, Cygnus Technology). Evoked IPSCs were elicited in the presence of 10 μM DNQX, 40 μM APV, and CGP 36216 hydrochloride (Tocris Bioscience) using the bipolar stimulating electrode placed within 250 μm of the recorded cell soma in the CA1 stratum pyramidale (proximal stimulation for perisomatic inhibition). The rectangle current pulses (duration: 200 μs, frequency: 20 Hz) were delivered for evoked IPSCs. The paired pulse stimuli were carried out by delivering a pair of stimuli with an interval of 100 ms. The amplitudes of the evoked responses were measured, and the paired‐pulse ratio (PPR) was calculated as the ratio of the two responses (second/first). Data from EGFR‐IUE mice with increased astrogenesis lower than 20% compared to Ctrl‐IUE mice were excluded.

### 2.7 Western blotting

Microdissected CA1 pyramidal cell layer from hippocampi was homogenized using a chilled Vibrahomogenizer (Vibra cell, SONICS) containing 100 μl of RIPA buffer [1% Triton X‐100, 0.1% SDS, 150 mM NaCl, 2 mM EDTA, 50 mM NaF, 10 mM sodium pyrophosphate, 1.0 mM Na_3_VO_4_, 1.0 mM PMSF, and complete protease inhibitor cocktail (Roche)]. The lysate was then centrifuged at 12,000*g* for 20 min at 4°C and the supernatant was collected for Western blot analysis. The protein concentration was determined using a Pierce™ BCA Protein Assay Kit (ThermoFisher Scientific 23225) and the tubes were stored at −20°C. 10% SDS‐PAGE was used to separate protein samples. After electrophoresis, the gels were transferred to polyvinylidene fluoride (PVDF) membranes (Millipore) using a constant voltage of 300 mA for 90 min. The membranes were then blocked in 5% milk in TBST (25 mM Tris–HCl, 150 mM NaCl, and 0.1% Tween 20, pH 7.4) for one half hour at RT on a rocker and incubated in the primary antibody overnight on a rocker at 4°C. Primary antibodies were made with 5% milk in TBST solutions [rabbit anti‐gephyrin (1:1000; Cell Signaling Technology 14304); mouse anti‐PSD95 (1:500; Sigma‐Aldrich MAB1596); mouse anti‐β‐actin (1:5000; HUABIO M1210‐2)]. Following primary antibody incubation, the membranes were rinsed three times in TBST before being incubated with anti‐mouse horseradish peroxidase (HRP)‐conjugated secondary antibody (1:10,000 in 5% milk in TBST; ThermoFisher Scientific 31430) and anti‐rabbit HRP‐conjugated secondary antibody (1:10,000; ThermoFisher Scientific 31460) for 1 h on a rocker at RT. After being washed in TBST three times on a rocker at RT, protein bands were then visualized using ECL‐Plus Western blotting detection reagents (ThermoFisher Scientific 1863096 and 1863097). Quantification of the intensity of the protein bands obtained in Western blots was performed using the NIH ImageJ software and results were normalized to the appropriate β‐actin bands.

### 2.8 Intra‐CA1 injection procedures

Stereotaxic surgical procedures were followed as described previously (Li et al., 2016). Data from EGFR‐IUE mice with increased astrogenesis lower than 20% compared to Ctrl‐IUE mice were excluded. All surgical instruments were sterilized. The IUE mice or vehicle C57BL/6J mice at P30–35 were anesthetized with isoflurane (3% for induction, 1.5%–2.0% thereafter) in a stereotaxic apparatus (D01453‐003, RWD Life Science, China). The position of the head was horizontally adjusted. A stereo microscope was used to observe the small incision exposed above the skull. All soft tissues from the surface of the skull were removed and the skull was cleaned. The guide cannulae (I.D. 0.34 mm; 62060; RWD Life Science, China) were implanted (bilaterally) 1 mm above the intended site of injection according to the atlas of the mouse brain (Paxinos & Franklin, 2012). Stereotaxic coordinates for the CA1 regions of the dorsal hippocampus were AP: −1.82 mm from bregma, *L*: ±1.05 from the sagittal suture and *V*: −1.5 mm from the skull surface. The implanted cannulae were anchored to the skull with metal screws and secured by dental cement. Stainless steel stylets (O.D. 0.30 mm; 62131; RWD Life Science, China) were inserted into the guide cannulae to keep them free of debris. All mice were allowed to recover from the surgery for at least 7 days.

For drug infusion, the stylets were removed from the guide cannulas and replaced by 32‐gauge microinjection needles (1 mm below the tip of the guide cannulas). The injector cannula was attached to a polyethylene tube fitted to a 1‐μl Hamilton syringe. The injection solution (0.125 μg BMI for IUE mice, 0.25 μg or 0.5 μg clonazepam for vehicle mice) or ACSF (as Ctrl) was administered manually at a total volume of 1 μl/mouse (0.5 μl in each side, intra‐CA1) over a period of 30 s in mice daily at 30 min before the behavioral tests or electrophysiological recordings. Injection needles were left in place for an additional 60 s to ensure drug infusion.

### 2.9 In vivo electroencephalogram (EEG) recording and analysis

The electrodes (795500, diameter 0.125 mm; A.M. Systems) were implanted into the right ventral hippocampus of IUE mice (AP: −1.82 mm, *L*: ±1.15 mm, *V*: −1.5 mm) for EEG recording (Wang et al., 2017), and the guide cannulae (I.D. 0.34 mm; 62060; RWD Life Science, China) were implanted (bilaterally) as mentioned in Section 2.8 for intra‐CA1 injection of BMI or ACSF.

After recovery (at least 7 days), EEGs were recorded 30 min after ACSF or BMI administration over a period of 25 min with a Neuroscan system (NuAmps, Neuroscan System) in IUE mice. EEGs were processed and the EEG power for different frequency bands was determined with the Neuroarchiver tool (Open Source Instruments). EEG epochs were also exported into LabVIEW (National Instruments) for coastline analysis. Glitches were excluded if they exceeded 5 × the root mean square of the baseline noise, and the signal was high‐pass filtered at 10 Hz before calculating the coastline as the sum of the absolute difference between successive points. Burst, coastline, and EEG high‐frequency power analysis were averaged over 25 min (Wykes et al., 2012). Only the mice with correct locations of electrodes and viral expression were taken into analysis.

### 2.10 Statistics

SigmaPlot (Systat Software Inc.) and GraphPad Prism (Graph‐Pad Software Inc.) were used for data display and statistical analysis. We did not predetermine sample sizes. We used Kolmogorov–Smirnov normality test to determine most data are Gaussian distributed. Significance is reported as *p* < .05, and data were expressed as mean ± SEM. Two‐tailed Student *t*‐test (for normal distributed data), Mann–Whitney Rank Sum Test (for non‐normal distribution data), one‐way ANOVA followed by a post hoc multiple comparison analysis based on the Bonferroni test, or two‐way ANOVA followed by a post hoc multiple comparison analysis based on the Holm–Sidak test were used to determine significant levels between treatments and controls. Distributions of mEPSC and mIPSC amplitudes and interevent intervals were compared using Kolmogorov–Smirnov test.

## 3 RESULTS

### 3.1 Increasing astrogenesis during development in the hippocampus impairs social interaction and increases repetitive behavior in EGFR‐IUE mice

**FIGURE 1:**
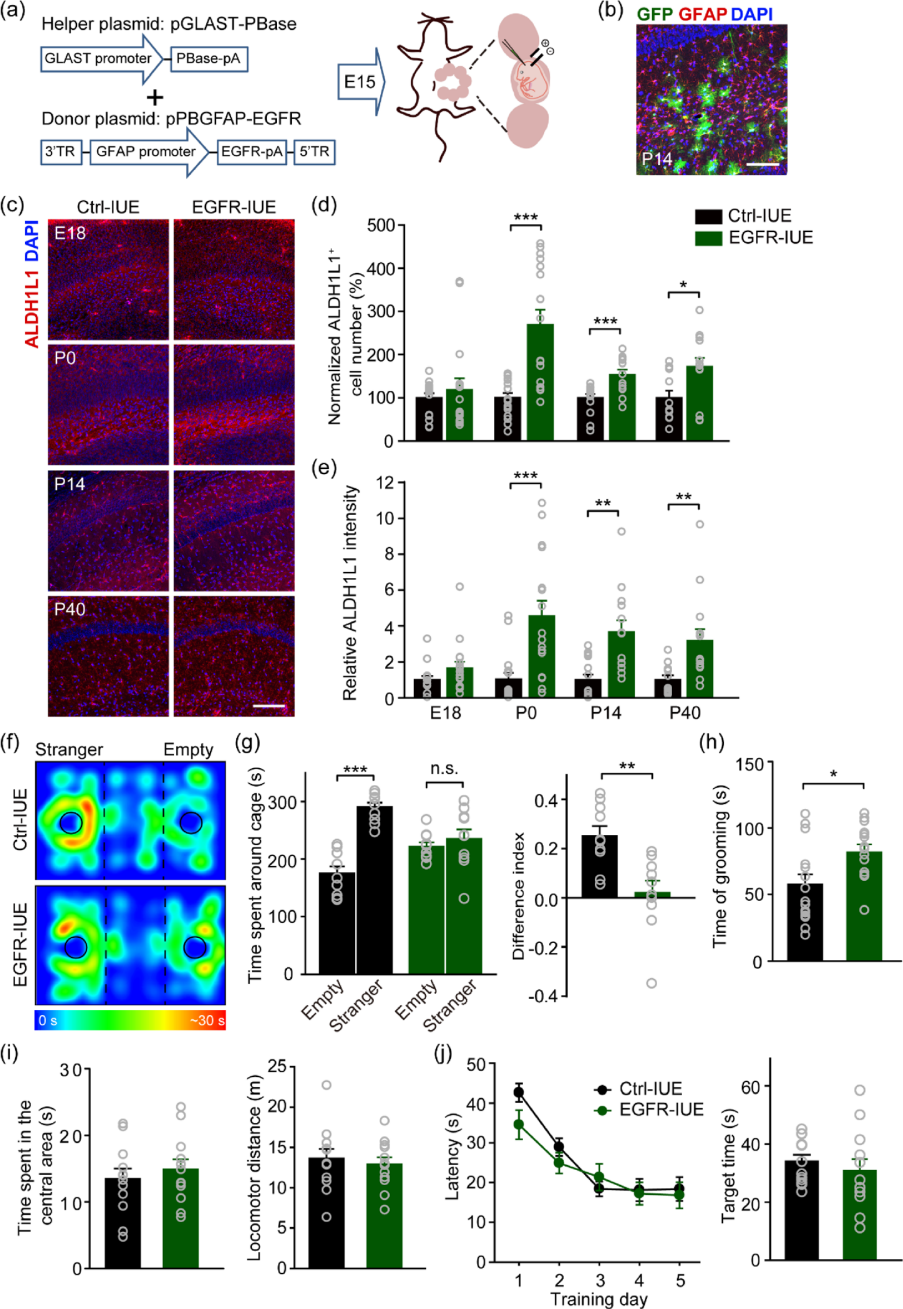
Increasing astrogenesis during development in the hippocampus impairs social interaction and increases repetitive behavior in EGFR‐IUE mice. (a) Schematics of the experimental procedures of IUE. (b) Representative images from P14 IUE mice transfected with test plasmids (pGLAST‐PBase and pPBGFAP‐eGFP) in the hippocampus immunohistostained with GFAP. Scale bar, 100 μm. (c–e) Representative images of immunohistostaining of ALDH1L1 (c), quantification of normalized ALDH1L1‐positive (ALDH1L1^+^) cell number (d), and relative ALDH1L1 intensity (e) in hippocampal CA1 of IUE mice at E18, P0, P14, and P40. Scale bar, 100 μm. *n* = 11–16 slices/4 mice per group. (f, g) Representative heat maps of animal positions (f) and quantification of time spent in close proximity to the cages (g, left) and the difference index (g, right) in the three‐chamber social interaction test. *n* = 10 per group. (h) Quantification of time spent grooming in Ctrl‐IUE and EGFR‐IUE mice. *n* = 15 per group. (i) Quantification of time spent in the central area (left) and distance traveled (right) in the open field test. *n* = 12 per group. (j) Quantification of escape latency in each session of the hidden‐platform test (left) and target quadrant searching time in the probe test (right) in the MWM task. *n* = 12 per group. Data are mean ± SEM. ^*^ *p* < .05, ^**^ *p* < .01, ^***^ *p* < .001 (in d E18, d P0, and e, Mann–Whitney rank sum test; in g left, one‐way ANOVA with post hoc Bonferroni *t*‐test; in d P14, d P40, g right, h, i, and j right, *t*‐test; in j left, two‐way ANOVA with post hoc Holm–Sidak test)

To investigate the role of aberrant astrocyte development in ASD pathogenesis, we generated a mouse model with increased hippocampal astrogenesis during early development by overexpressing EGFRs in astrocytes using IUE. IUE is a relatively efficient method for delivering plasmid DNA into progenitors in the brain of defined regions in vivo (Bai et al., 2003; Chen & LoTurco, 2012; Manent et al., 2009). Meanwhile, EGFR transgenesis in progenitor cells has been used to trigger cell differentiation into astrocytes and the proliferation of astrocytes at a later developmental stage (Burrows et al., 1997; Chen & LoTurco, 2012; Sun et al., 2005). Hence, we performed IUE with a set of *piggyBac* plasmids in mice at E15 as previously described (Shen et al., 2016) to yield preferential EGFR transfection in CA1 subfield (Navarro‐Quiroga et al., 2007; Figure 1a), because late gestation is a critical period for CA1 development (Tole & Grove, 2001). The GLAST promoter in pGLAST‐PBase plasmid helps to direct expression of transposase in GLAST‐positive progenitors, and the GFAP promoter in pPBGFAP‐EGFR (EGFR‐IUE; pPBGFAP as Ctrl‐IUE and pPBGFAP‐eGFP as test) plasmid will further encode EGFR selectively in GFAP‐positive progenitors. This strategy resulted in GFP‐positive cells in the hippocampus that were also GFAP positive in the test group (Figure 1b), confirming the transfected cells are developing astrocytes. Compared to Ctrl‐IUE mice, the notably increased relative EGFR intensity detected in ALDH1L1‐positive cells in hippocampal CA1 from EGFR‐IUE mice at postnatal day 0 (P0; Figure S1a,b) indicated the feasibility of using this method to achieve efficient and stable EGFR overexpression in astrocytes.

We then examined whether and when EGFR overexpression induces excessive astrogenesis in hippocampal CA1. We found a significant increase in the ALDH1L1‐positive cell number and fluorescence intensity in CA1 from P0 EGFR‐IUE mice, and such an increase was sustained throughout postnatal development as shown in P14 and P40 mice (Figure 1c–e), implying that the increased hippocampal astrogenesis is a long‐lasting phenomenon. Similar results were observed with immunostaining for GFAP (Figure S1c–e). In addition, unaltered numbers of processes and ramification of astrocytes in hippocampal CA1 of P14 EGFR‐IUE mice were observed in comparison with Ctrl‐IUE mice (Figure S1f–i), indicating that EGFR overexpression by IUE at E15 does not alter the morphology of astrocytes. Moreover, an increased number of Ki67 positive cells co‐labeled with ALDH1L1 (Figure S1j,k) further confirmed increased astrocyte lineage proliferation in neonatal hippocampal CA1. However, immunostaining for HuC/HuD and Iba‐1 showed unaltered neurogenesis and microglia activation in P0 EGFR‐IUE mice, respectively (Figure S2). These data indicate that EGFR overexpression in the developing astrocytes in late gestation promotes stable astrogenesis in hippocampal CA1 of neonatal EGFR‐IUE mice.

We next investigated the effects of increased hippocampal astrogenesis on autistic‐like behavior in mice. Impaired social interaction is a hallmark of autism. We assessed a measure of this trait in 5–6‐week‐old IUE mice, using a three‐chamber social interaction test (Ren et al., 2020; Smith et al., 2011) in which mice are first acclimated to the arena and then choose between a social chamber containing a caged and sex‐ and age‐matched C57BL/6J stranger mouse and a chamber with an empty cage. Compared to Ctrl‐IUE mice, EGFR‐IUE mice failed to show a social preference in either measure as they explored the empty and mouse‐occupied cages equally, exhibiting social interaction deficits (Figure 1f,g). Another hallmark autistic trait is repetitive, stereotyped behaviors such as body rocking, hand flapping, and self‐injurious behavior. These behaviors have been assumed to be equivalent to repetitive self‐grooming in mice (Ren et al., 2020; Smith et al., 2011). Compared to controls, EGFR‐IUE mice exhibited significantly high self‐grooming scores (Figure 1h). However, they displayed normal levels of anxiety in the OFT (Figure 1i) and intact spatial learning and memory in the MWM task (Figure 1j). Virtually no differences in the general development (including body length, body weight, and overall hippocampal morphology), the gross appearance of the brain or H&E staining of the hippocampus was observed among vehicle (Ctrl; sex‐ and age‐matched C57BL/6J mice), Ctrl‐IUE, and EGFR‐IUE mice (Figure S3a–e). Meanwhile, similar results in the behavioral tests (social interaction, repetitive self‐grooming, OFT, and MWM test) were also found between Ctrl and Ctrl‐IUE mice (Figure S3f–j). These data showed that increasing hippocampal astrogenesis during development contributed to impaired social interaction and increased repetitive behavior in mice.

### 3.2 Enhanced inhibitory synaptic transmission in EGFR‐IUE mice

**FIGURE 2:**
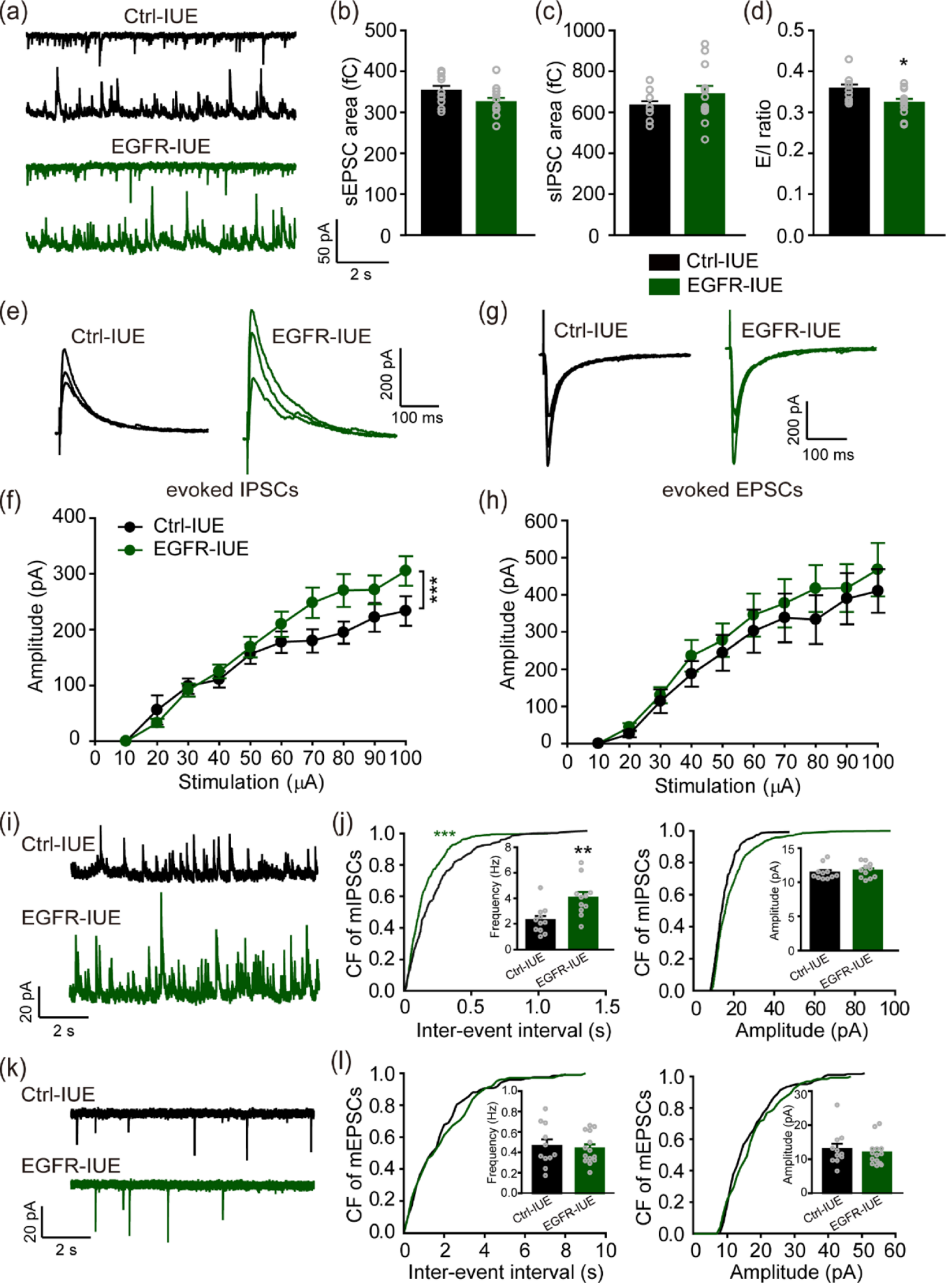
Enhanced GABAergic synaptic transmission in EGFR‐IUE mice. (a) Representative traces of sEPSCs (top) and sIPSCs (bottom), respectively, recorded at −70 and 10 mV in a CA1 pyramidal cell of Ctrl‐IUE (black) and EGFR‐IUE (green) mice. (b–d) Quantification of sEPSC (b) and sIPSC (c) charge transfers and *E*/*I* ratios (d) in Ctrl‐IUE and EGFR‐IUE mice. *n* = 10–12 slices/3 mice per group. (e–h) Sample superimposed traces of evoked IPSCs (e) and EPSCs (g) at stimulus intensity of 30, 60, and 90 μA and quantification of evoked IPSC (f) and EPSC (h) amplitudes in Ctrl‐IUE and EGFR‐IUE mice. *n* = 16–20 slices/3 mice per group for (f) and 9–13 slices/3 mice per group for (h). (i–l) Representative traces of mIPSCs (i) and mEPSCs (k) and cumulative frequency (CF) plots of interevent interval (left) and amplitude (right) of mIPSCs (j) and mEPSCs (l) in Ctrl‐IUE and EGFR‐IUE mice. *n* = 11 slices/3 mice per group for (j) and 11–14 slices/3 mice per group for (l). Data are mean ± SEM. ^*^ *p* < .05, ^**^ *p* < .01, ^***^ *p* < .001 (in b, c, d, and j inset, *t*‐test; in f and h, two‐way ANOVA with post hoc Holm–Sidak test; in j and l, two‐sample Kolmogorov–Smirnov test; in l insert, Mann–Whitney rank sum test)

A widely accepted hypothesis on ASD etiology proposed that *E*/*I* imbalance is a main pathophysiological mechanism that underlies the social, behavioral, emotional, cognitive, and sensorimotor abnormalities (Etherton et al., 2011; Sohal & Rubenstein, 2019). *E*/*I* imbalance is due primarily to abnormal glutamatergic and GABAergic neurotransmission in key brain regions including the hippocampus. We thus hypothesized that increasing neonatal astrogenesis may alter *E*/*I* ratio by affecting excitatory and/or inhibitory synaptic transmission in hippocampal CA1. To directly compare glutamatergic and GABAergic synapses on a cell‐by‐cell basis, we recorded sEPSCs and sIPSCs by alternately clamping at AMPA or GABA_A_ receptor channel reversal potential (Zhou et al., 2009) in CA1 pyramidal neurons. The sEPSC charge transfer was slightly but not significantly lower (Figure 2a,b) whereas the sIPSC charge transfer was slightly but not significantly higher (Figure 2a,c), causing a significant reduction in *E*/*I* ratio (Figure 2d) in EGFR‐IUE mice. Similar results in sEPSC charge transfer and sIPSC charge transfer were also observed in Ctrl‐IUE and EGFR‐IUE mice under pharmacological isolation (Figure S4), verifying that sEPSC and sIPSC responses recorded at the reversal potential and under pharmacological isolation are identical. We next recorded evoked IPSCs and evoked EPSCs in CA1 pyramidal neurons by directly stimulating the CA1 pyramidal layer (perisomatic inhibition) and the Shaffer collateral (SC), respectively. The amplitude of evoked IPSCs, but not of evoked EPSCs, was strongly augmented in EGFR‐IUE mice (Figure 2e–h). We then recorded mEPSCs and mIPSCs in CA1 pyramidal neurons. We found significantly increased mIPSC frequency but not amplitude in P14 EGFR‐IUE mice in comparison with controls (Figure 2i,j; Table S1), whereas neither the frequency nor the amplitude of mEPSCs in EGFR‐IUE mice was changed (Figure 2k,l; Table S1). Similar results of *E*/*I* ratio, inhibitory and excitatory synaptic responses were also found in P40 EGFR‐IUE mice (Table S2), indicating that such an enhancement of GABAergic inhibition in CA1 pyramidal neurons persists into adulthood. In addition, no change was found in either excitatory or inhibitory responses in CA1 pyramidal neurons from Ctrl and Ctrl‐IUE mice (Table S3). Together, these data indicate that increasing astrogenesis in the hippocampus during development induces a reduction in *E*/*I* ratio via enhancing GABAergic synaptic transmission in CA1 pyramidal neurons of EGFR‐IUE mice.

### 3.3 Increased inhibitory synapse formation in EGFR‐IUE mice

**FIGURE 3:**
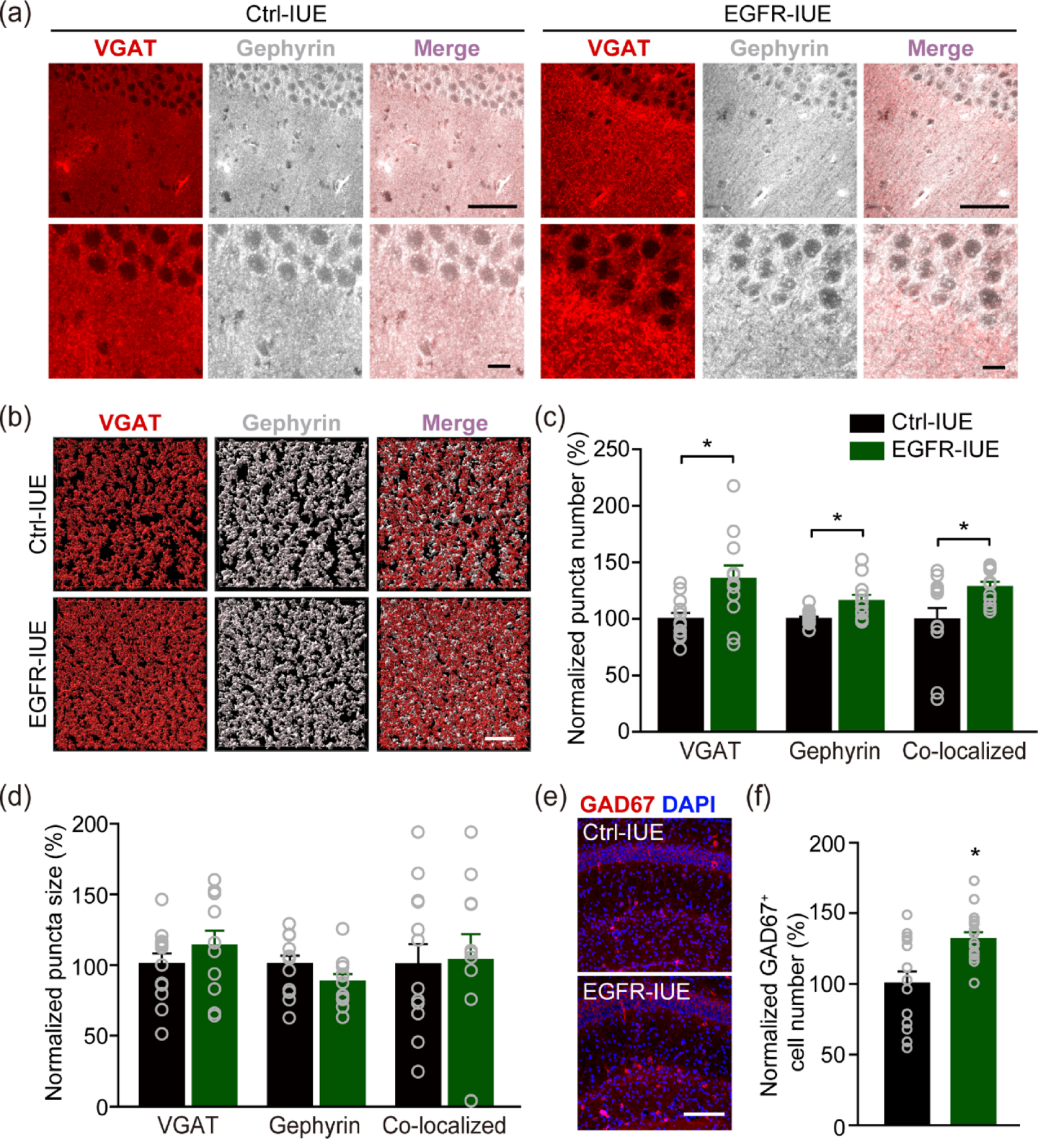
Increased inhibitory synapse formation in EGFR‐IUE mice. (a, b) Representative images (a) and reconstructions (b) of immunohistostaining of VGAT, gephyrin, and VGAT/gephyrin co‐localized puncta in hippocampal CA1 of Ctrl‐IUE and EGFR‐IUE mice. Scale bars, 50 and 10 μm (a), 3 μm (b). (c, d) quantification of the number (c) and size (d) of VGAT, gephyrin and VGAT/gephyrin co‐localized puncta in hippocampal CA1 of Ctrl‐IUE and EGFR‐IUE mice. *n* = 11–12 slices/4 mice per group. (e, f) Representative images of immunostaining (e) and quantification of the number of GAD67‐positive cells (f) in hippocampal CA1 (SR and SLM) of Ctrl‐IUE and EGFR‐IUE mice. Scale bar, 100 μm. *n* = 14–16 slices/4 mice per group. Data are mean ± SEM. ^*^ *p* < .05 (in c VGAT, d VGAT, d gephyrin, and d co‐localized, *t*‐test; in c gephyrin and c co‐localized, Mann–Whitney rank sum test)

The increased mIPSC frequency in CA1 pyramidal neurons in EGFR‐IUE mice implicates that enhanced hippocampal astrogenesis during development may have presynaptic effects on GABAergic inhibitory transmission in the hippocampus. An increase in the number of inhibitory synapses formed onto CA1 pyramidal neurons may account for the increased mIPSC frequency. We thus first analyzed the levels of postsynaptic proteins in Ctrl‐IUE and EGFR‐IUE mice. We found an increase in the total protein level of gephyrin (for inhibitory synapses) with unaltered total protein level of PSD95 (for excitatory synapses) in EGFR‐IUE mice (Figure S5), suggesting a selective increase in the density of inhibitory synapses in hippocampal CA1. We then measured the density of synapses using antibodies against synaptic vesicle proteins and postsynaptic proteins. Analysis of magnified images from the CA1 revealed notable increases in the numbers of vesicular GABA transporter (VGAT, a marker for inhibitory synapses), gephyrin, and VGAT/gephyrin co‐localized puncta with unaltered size of puncta in EGFR‐IUE mice (Figure 3a–d), indicating an increase in the inhibitory synapse formation in the hippocampal CA1. Meanwhile, the numbers of inhibitory synapses in CA3 and cortical layer VI areas were not significantly increased in EGFR‐IUE mice (Figure S6). In contrast, no changes in the number and size of vesicular glutamate‐transporter 1 (VGlut1, a marker for excitatory synapses) puncta were observed in the CA1 (Figure S7a–c). Furthermore, an increase in the glutamic acid decarboxylase 67 (GAD67, for GABAergic interneurons)‐positive cell number (Figure 3e,f) was also detected in CA1 area of the EGFR‐IUE mice, whereas the intensity of Ca^2+^/calmodulin‐activated kinase II (CaMKII, for glutamatergic neurons; Figure S7d,e) did not change. An increase in the frequency of miniature synaptic currents may also reflect a rise in the presynaptic release probability. This is supported by a reduction of the PPR of evoked IPSCs in EGFR‐IUE mice (Figure S8a–d), as the degree of the PPR is inversely related to the probability of neurotransmitter release (He et al., 2019). Together, these data imply that excessive astrogenesis during development promoted inhibitory synaptic transmission in the hippocampus via presynaptic mechanisms.

### 3.4 Suppressing the enhanced GABAergic inhibition rescues autistic‐like behavior in EGFR‐IUE mice

Does localized increased GABAergic activity in the hippocampus is sufficient and necessary to induce ASD‐like behavioral traits? To address this question, we first tested the effect of localized clonazepam (a long‐acting, traditional benzodiazepine) in the hippocampus on ASD‐like behavior in vehicle mice. We found that vehicle C57BL/6J mice received clonazepam treatment (intra‐CA1 injection; 0.5 μg/1 μl/mouse, 0.5 μl each side) displayed impaired social interaction and increased repetitive self‐grooming compared to those injected with ACSF (Figure S9). Such an effect of clonazepam was dose‐dependent, as mice received a lower dose of clonazepam (0.25 μg/1 μl/mouse, 0.5 μl each side) displayed only social interaction deficits with unaltered repetitive self‐grooming scores (Figure S9). These data indicate that localized increased GABAergic activity in the hippocampus by clonazepam is sufficient to induce ASD‐like behavioral traits in mice.

**FIGURE 4:**
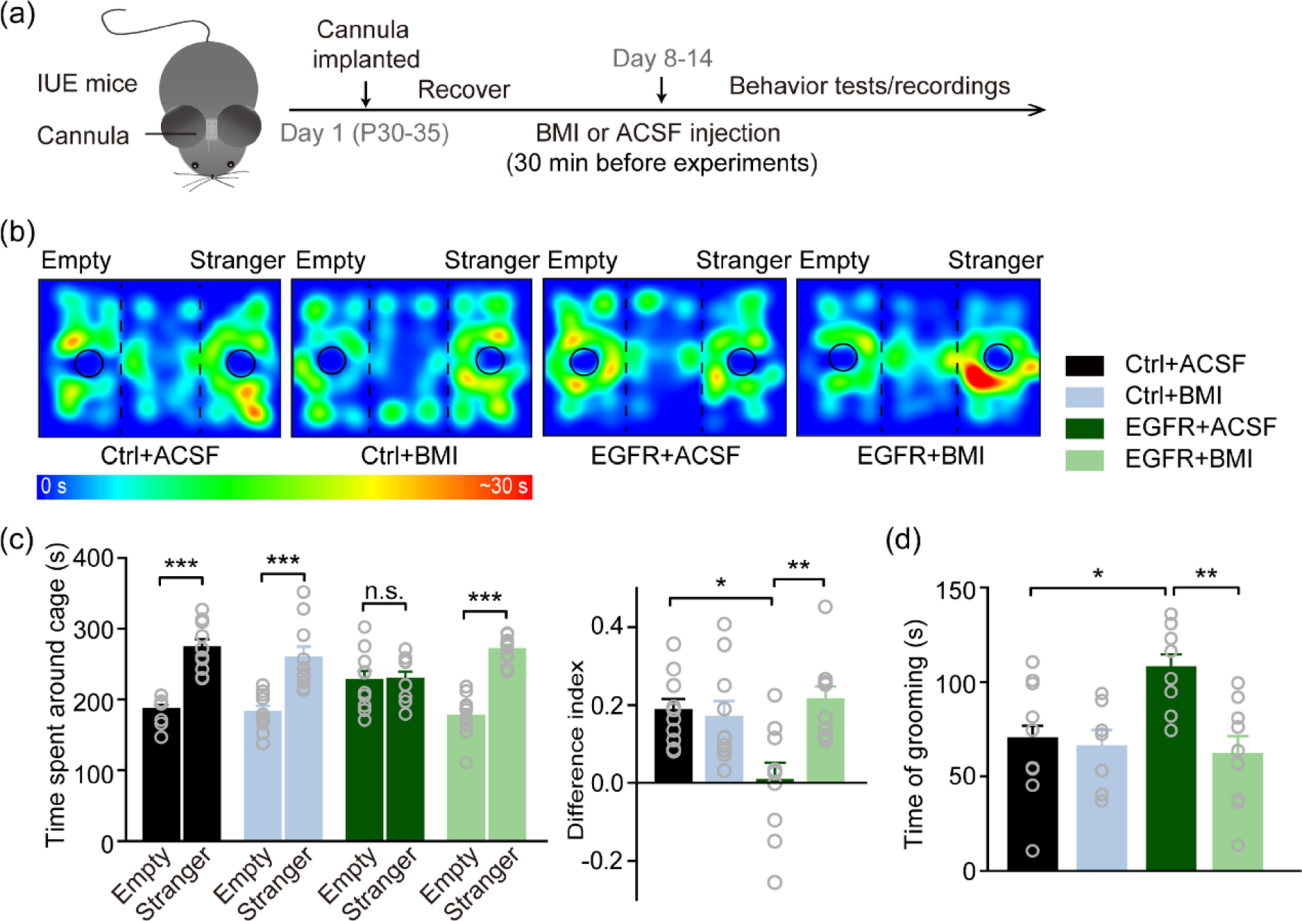
Suppressing enhanced GABAergic inhibition rescues autistic‐like behavior in EGFR‐IUE mice. (a) Schematics of the experimental procedures of intra‐CA1 BMI or ACSF administration. (b, c) Representative heat maps of animal positions (b) and quantification of time spent in close proximity to the cages (c, left) and the difference index (c, right) in the three‐chamber social interaction test. *n* = 10 per group. (d) Quantification of time spent grooming in BMI‐ or ACSF‐infused Ctrl‐IUE (Ctrl‐BMI or Ctrl‐ACSF) and EGFR‐IUE (EGFR‐BMI or EGFR‐ACSF) mice. *n* = 7–9 per group. Data are mean ± SEM. ^*^ *p* < .05, ^**^ *p* < .01, ^***^ *p* < .001 (one‐way ANOVA with post hoc Bonferroni *t*‐test)

**FIGURE 5:**
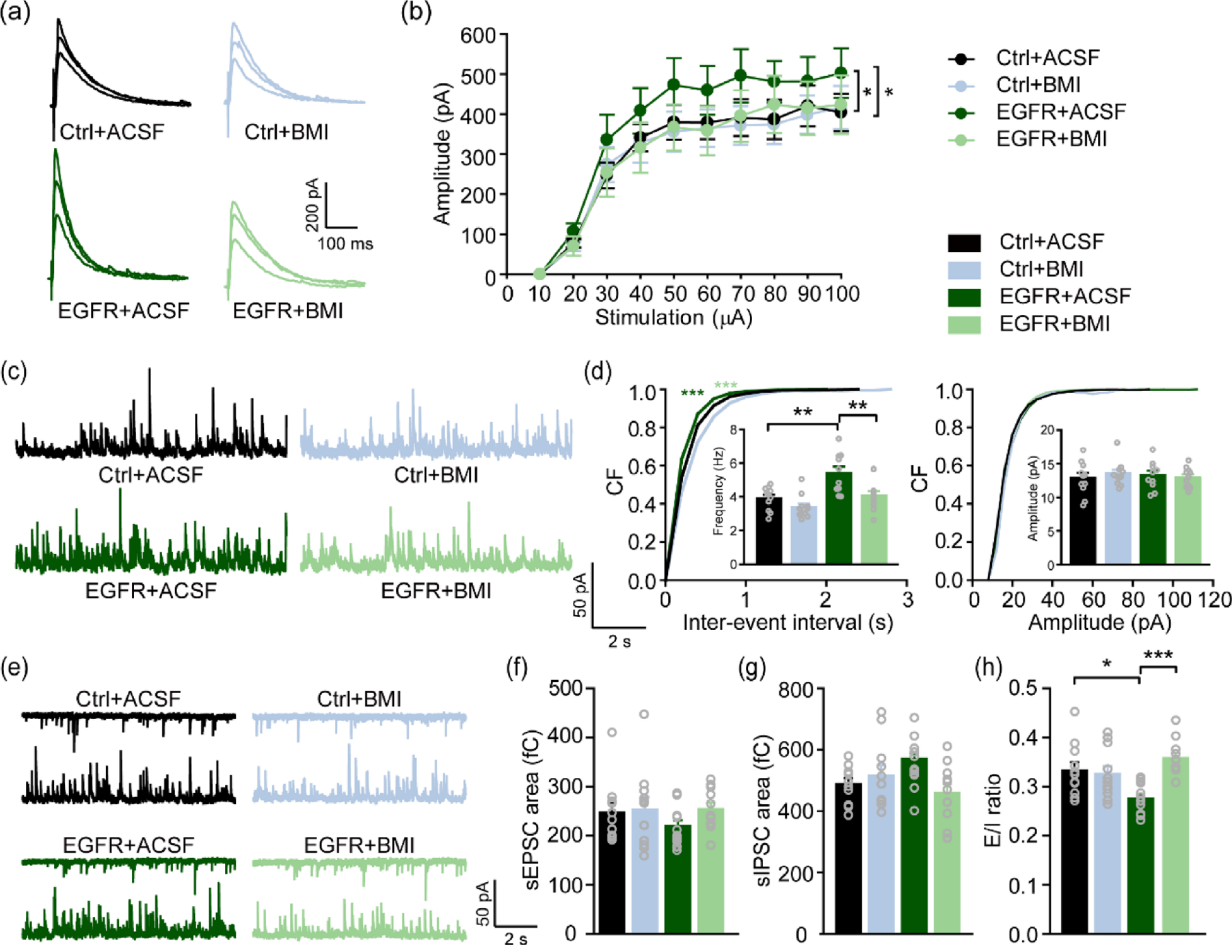
Suppressing enhanced GABAergic inhibition restores hippocampal *E*/*I* balance in EGFR‐IUE mice. (a, b) Sample superimposed traces of evoked IPSCs at stimulus intensity of 30, 60, and 90 μA (a) and quantification of evoked IPSC amplitude (b) in BMI‐ or ACSF‐infused Ctrl‐IUE and EGFR‐IUE mice. *n* = 10–12 slices/3 mice per group. (c, d) representative traces of mIPSCs (c) and CF plots (d) of mIPSC interevent interval (left) and amplitude (right) in BMI‐ or ACSF‐infused Ctrl ‐IUE and EGFR‐IUE mice. *n* = 10–11 slices/3 mice per group. (e) Representative traces of sEPSCs (top) and sIPSCs (bottom), respectively, recorded at −70 mV and 10 mV (bottom) in a single CA1 pyramidal neuron of BMI‐ or ACSF‐treated Ctrl‐IUE and EGFR‐IUE mice. (f–h) Quantification of sEPSC (f) and sIPSC (g) charge transfers and *E*/*I* ratios (h) in BMI‐ or ACSF‐treated Ctrl‐IUE and EGFR‐IUE mice. *n* = 11–12 slices/3 mice per group. Data are mean ± SEM. ^*^ *p* < .05, ^**^ *p* < .01, ^***^ *p* < .001 (in b, two‐way ANOVA with post hoc Holm–Sidak test; in d, two‐sample Kolmogorov–Smirnov test; in d insets, f, g, and h, one‐way ANOVA with post hoc Bonferroni *t*‐test)

Lastly, we performed rescue experiments by intra‐CA1 injection of (BMI; a GABA_A_ receptor antagonist; Figure 4a) to verify if the enhanced GABAergic inhibition following increased astrogenesis in hippocampal CA1 was responsible for autistic‐like behavior in EGFR‐IUE mice. EGFR‐IUE mice received BMI treatment (0.125 μg/1 μl/mouse, 0.5 μl each side) displayed improved social interactions (Figure 4b,c) and reduced repetitive self‐grooming scores (Figure 4d) compared to those injected with ACSF. BMI treatment also attenuated the increased amplitude of evoked IPSCs (Figure 5a,b) and mIPSC frequency (Figure 5c,d) in EGFR‐IUE mice. Although the decreased PPR was not rescued (Figure S8e,f) by BMI, the reduced *E*/*I* ratio in EGFR‐IUE mice returned to the normal level observed in Ctrl‐IUE mice (Figure 5e–h). Neither the behavioral consequences nor the GABAergic neurotransmission in Ctrl‐IUE mice was affected by BMI (Figures 4b–d and 5a–h). Besides, intra‐CA1 injection of BMI did not induce local seizure activity and affect excitatory network activity in the hippocampus of Ctrl‐IUE and EGFR‐IUE mice (Figure S10), indicating that the behavioral recovery in EGFR‐IUE mice by BMI is not due to bursting excitatory network activity in the hippocampus. Thus, suppressing the aberrantly enhanced GABAergic inhibition by a right dose of BMI could rescue autistic‐like behavior and restore hippocampal *E*/*I* balance in EGFR‐IUE mice.

## 4 DISCUSSION

In the current study, we present evidence that increasing astrogenesis during development in the hippocampus produces autistic‐like behavior in mice and decreased *E*/*I* ratio in CA1 area of the hippocampus. Enhancement of GABAergic neurotransmission, which involves increases in presynaptic GABA release probability and inhibitory synapse formation on CA1 pyramidal neurons, underlies this *E*/*I* imbalance. Enhanced inhibitory transmission in the hippocampus is causally linked to abundant hippocampal astrogenesis‐induced autistic‐like traits in mice as suppressing the aberrantly enhanced GABAergic inhibition rescues these traits and restores the *E*/*I* balance. The study reveals a developmental role of astrocytes in producing the behavioral phenotypes of ASD, supporting the GABAergic neurotransmission may serve as an effective therapeutic target for treating ASD.

ASD is a multistage, progressive disorder of brain development, spanning from prenatal to early postnatal developmental stages (Courchesne et al., 2019). The development of functional neural circuits relies upon coordination of various cell types, thus adequate neurogenesis and gliogenesis are required for proper neural circuit formation (Sloan & Barres, 2014). Disruptions in any of the mechanisms that affect neurogenesis and astrogenesis may lead to perturbations in the relative ratios of neurons and astrocytes, consequently resulting in inappropriate neuronal connections and networks (Sloan & Barres, 2014). Some neurodevelopmental diseases such as Noonan syndrome (Tartaglia et al., 2001), Neurofibromatosis‐1 (Hegedus et al., 2007), Down syndrome (Zdaniuk et al., 2011), have been shown to have abnormal astrocyte development and proliferation. Our results implicate that abnormal astrocyte development may also lead to ASD. There is evidence suggesting that aberrant astrocyte function leads to ASD. A recent study has identified increased CD8+ cytotoxic T‐lymphocytes and cytotoxic debri “blebs” derived from astrocytes in more than half of autism postmortem brain (DiStasio et al., 2019). In addition, immune activation, especially maternal immune activation (MIA), is considered as a primary risk factor for ASD (Courchesne et al., 2019; Estes & McAllister, 2016; Gluckman et al., 2008). As the onset of astrogenesis is a temporally regulated process that relies upon exogenously secreted cues and intrinsic chromatin changes (He et al., 2005; Laug et al., 2018; Nakashima et al., 1999), the predisposing risk factors such as immune activation may impair astrocyte development and functions, which are likely to strongly affect neural circuit formation, leading to abnormal network activity. Indeed, fetuses of lipopolysaccharide (LPS)‐treated dams have shown astrogliosis, extensive cell death and an increased number of cells producing TNF‐α (Chua et al., 2012), and exhibited more severity of experimental autoimmune encephalomyelitis in adult offspring with parallel exacerbated proliferation in microglia and astrocytes (Zager et al., 2015). It has also been reported that astrocytes exposed with maternally derived cytokines after MIA seem to secrete various cytokines that affect brain development (Ibi et al., 2013; Yamada et al., 2014). It will be interesting to investigate how immune activation leads to ASD via astrocyte‐mediated circuit rearrangement in future studies.

Imbalanced *E*/*I* ratios have been reported in various brain areas in many ASD mouse models. Increased *E*/*I* ratios have been observed in mouse models of ASD (e.g., *Fmr1* ^−/y^ and *TSC* ^*+/−*^, Antoine et al., 2019; *MeCP2* ^−/−^, Chao et al., 2010; *Scn1a* ^*+/−*^, Han et al., 2012; *Shank3* ^−/−^, Peça et al., 2011). Reduced *E*/*I* ratios have been also reported in many ASD mouse models (e.g., *MeCP2* ^−/−^, Dani et al., 2005; *CNTNAP2* ^−/−^/*AHI1* ^−/−^, Sacai et al., 2020; *Scn2a* ^*+/−*^, Spratt et al., 2019; NL3 R451C, Tabuchi et al., 2007), similar to our EGFR‐IUE mice (Figure 2). These various causes of ASD have been shown to affect excitatory (Dani et al., 2005; Rhee et al., 2018; Smith et al., 2011; Spratt et al., 2019), inhibitory (Chao et al., 2010; Tabuchi et al., 2007; Vuong et al., 2018), or both (Antoine et al., 2019; Davenport et al., 2019; Han et al., 2014; Sacai et al., 2020) transmissions. In addition, studies in ASD patients have shown structural and functional changes in both glutamatergic excitatory (Purcell et al., 2001; Tang et al., 2014) and GABAergic inhibitory (Masuda et al., 2019; Robertson et al., 2016) circuits. The discrepancy in *E*/*I* balance in various models of ASD thus may result from differential changes in the relative activity of excitatory and inhibitory neurons in different brain areas (Sohal & Rubenstein, 2019). Therefore, *E*/*I* imbalance may represent a “final common pathway” (Sohal & Rubenstein, 2019) leading to disruptions of diverse network functions that often result in typical autistic traits.

Astrocyte‐mediated signaling events are well‐known to contribute to normal synapse development in the developing brain. In addition to the well‐studied astrocytic modulation of glutamatergic neurons and excitatory transmission in the brain, astrocyte‐mediated regulation of inhibitory circuits gains attention recently (Matos et al., 2018; Nguyen et al., 2020; Tsai et al., 2012), although the detailed mechanisms are still largely undefined. We show that the excessive astrogenesis enhances GABAergic neurotransmission, which is consistent with previous reports showing disrupted GABAergic inhibition in ASD (Cao et al., 2018; Chao et al., 2010; Fatemi et al., 2009; Tabuchi et al., 2007; Vuong et al., 2018). Similar to our results, astrocytes have been shown to promote GABAergic interneuron proliferation (Nguyen et al., 2020) and inhibitory synaptogenesis (Elmariah et al., 2005; Hughes et al., 2010), leading to enhanced GABAergic inhibition. Such effects on increased inhibitory synapse formation might be localized to the targeted CA1 area with highly increased astrogenesis. Interestingly, astrocytic activation by immune challenge in the second postnatal week selectively promotes excitatory synaptogenesis and enhances excitatory synaptic function in juvenile mice (Shen et al., 2016). The differences in the regulation of excitatory and inhibitory circuits by astrocytes could be due to the different developmental periods. It is also likely that astrocytes release different molecules that independently promote excitatory or inhibitory synapse formation (Clarke & Barres, 2013) at different developmental stages. Although we do not know the identity of the released molecule yet, it is unlikely thrombospondins (TSPs) contribute at this developmental stage, as TSPs secreted by astrocytes strongly induce excitatory (Christopherson et al., 2005) but not inhibitory (Hughes et al., 2010) synaptogenesis. Transforming growth factor beta 1 (TGF‐β1) secreted by astrocytes has been shown to promote inhibitory synapse formation in the neurons co‐cultured with astrocyte‐conditioned medium (ACM) (Diniz et al., 2014). In addition to the astrocyte‐secreted factors, it is also possible that astrocyte‐derived adhesion molecules affect inhibitory synapse formation through astrocyte‐neuron interaction (Nguyen et al., 2020) at this developmental stage. Thus, in early brain development, excessive astrogenesis may facilitate astrocyte‐derived factors that strongly induce the GABAergic synapse formation and increase the interneuron number (neurogenesis or migration). Further studies are required to clarify the mechanisms underlying astrocytic regulation of GABAergic inhibitory circuits in early development.

Our results show that moderate GABA_A_ receptor inhibition in hippocampal CA1 area by BMI effectively rescues autistic‐like behavior in EGFR‐IUE mice (Figure 4). However, low‐dose treatment with clonazepam has been shown to improve the social interaction deficit of BTBR (Han et al., 2014) and *Scn1a* ^*+/−*^ mice (Han et al., 2012). This could be due to different forms of *E*/*I* imbalance are involved in different ASD mouse models. Thus, balancing the *E*/*I* ratio in an unbalanced circuit may be the ultimate treatment strategy for a specific type of ASD. Indeed, a high dose of clonazepam (Figure S9) or zolpidem (α1‐subunit‐selective positive GABA_A_ receptor modulator) (Han et al., 2014) impairs sociability in C57BL/6J mice, supporting that tilted *E*/*I* balance truly underlies the behavioral phenotypes of ASD.

In conclusion, the current work depicts an important role of early hippocampal astrogenesis in promoting autistic‐like behavior in mice. Increasing hippocampal astrogenesis during early development tilts *E*/*I* balance via enhancing the GABAergic inhibition in the hippocampus, leading to typical autistic‐like traits in mice. As astrocytes modulate the development and function of excitatory and inhibitory synapses, understanding the molecular underpinnings of differential astrocyte‐mediated regulation of excitatory and inhibitory synapse development and function may provide essential insight into ASD pathology and better pharmacological and behavioral treatment strategies for ASD.

## CONFLICT OF INTEREST

The authors declare no conflict of interest.

## AUTHOR CONTRIBUTIONS

Yi Shen and Yu‐Dong Zhou conceived the study and coordinated the experiments. Juan Chen performed IUE and biochemical experiments. Juan Chen and Hua Wang performed plasmid constructions. Juan Chen and Xiao‐Lin Ma performed the behavioral experiments. Xiao‐Lin Ma performed electrophysiological recordings and data analysis. Hui Zhao, Xiao‐Yu Wang, Min‐Xin Xu, Hua Wang, and Cheng Peng performed the immunohistochemical experiments and data analysis. Xiao‐Lin Ma performed intra‐CA1 injection experiments. Juan Chen, Xiao‐Yu Wang, Tian‐Qi Yang, and Hua Wang performed data analysis of the behavior tests. Shuang‐Shuang Liu made constructive suggestions for imaging and puncta analysis. Man Huang modified the manuscript. Yi Shen and Yu‐Dong Zhou wrote the manuscript. All authors commented on the manuscript.

## Supporting information


**Appendix S1**: Supporting information.

## Data Availability

The data that support the findings of this study are available from the corresponding author upon reasonable request.
