# Supplementary material for: Increasing astrogenesis in the developing hippocampus induces autistic‐like behavior in mice via enhancing inhibitory synaptic transmission
Source: Glia. 2021 Sep 9;70(1):106–22. doi: 10.1002/glia.24091 (PMC9291003; doi:10.1002/glia.24091)
Supplement: Supplementary file 1 — Appendix S1: Supporting information. [file GLIA-70-106-s001.docx]

**Supporting Information – Chen et al.**

**Supplementary Figure 1:**

**
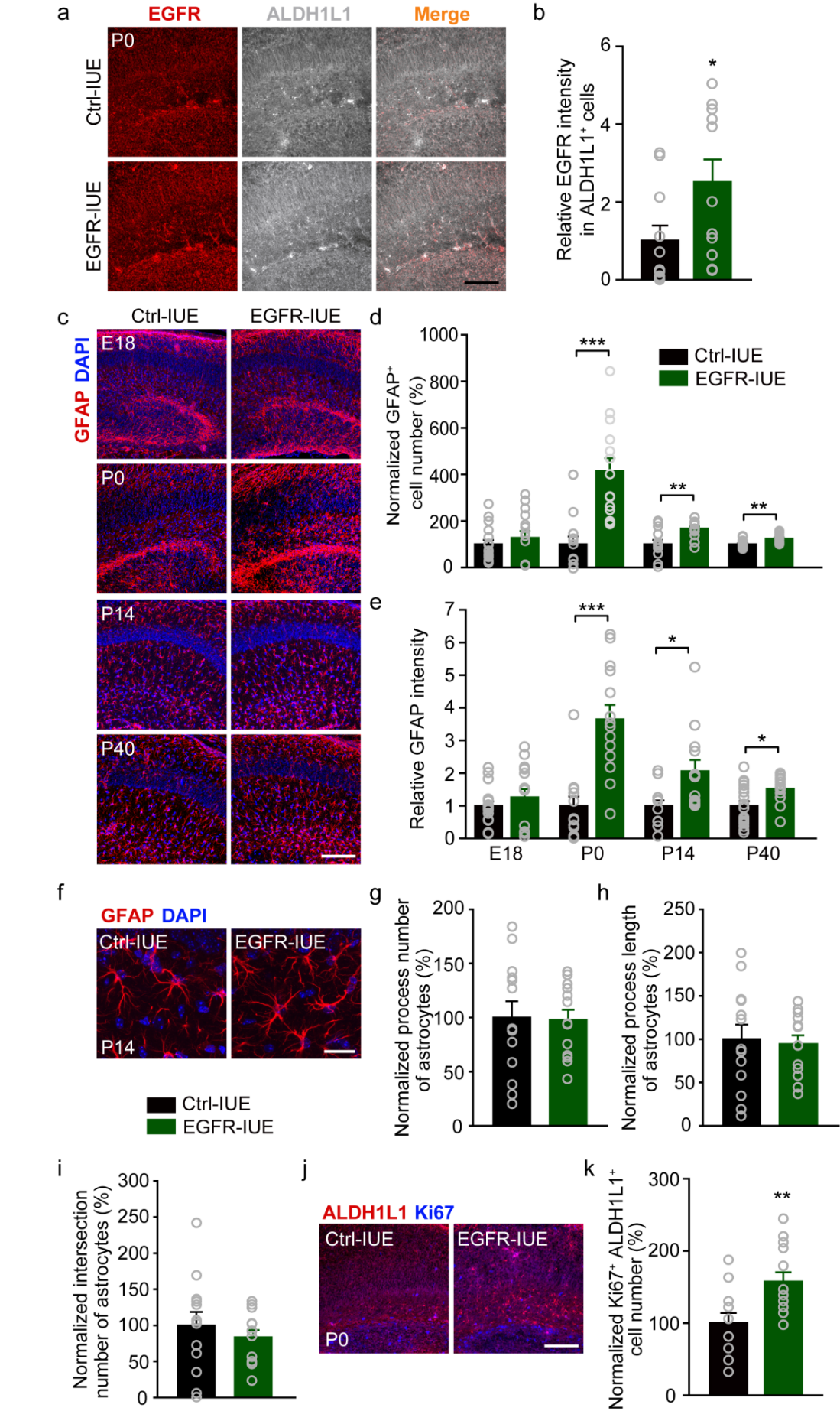
**

**Supplementary Figure 1 EGFR overexpression in developing astrocytes selectively promotes astrogenesis in CA1 area of the hippocampus.** Related to FIGURE 1. (**a, b**) Representative images of immunohistostaining of EGFR and ALDH1L1 (a) and quantification of relative EGFR intensity in ALDH1L1-positive (ALDH1L1^+^) cells (b) in hippocampal CA1 of IUE mice at P0. Scale bar = 100 μm. *n* = 11 slices/4 mice per group. (**c**-**e**) Representative images of immunohistostaining of GFAP (c) and quantification of normalized GFAP-positive (GFAP^+^) cell number (d) and relative GFAP intensity (e) in hippocampal CA1 of IUE mice at E18, P0, P14, and P40. Scale bar = 100 μm. *n* = 13-18 slices/4 mice per group. (**f-i**) Representative higher magnification images (f) and quantification of the normalized process number (g), process length (h), and intersection number (i) of GFAP^+^ astrocytes in hippocampal CA1 of IUE mice at P14. Scale bar = 25 μm. *n* = 13 slices/4 mice per group. (**j, k**) Representative images of immunohistostaining of ALDH1L1 and Ki67 (j) and quantification of co-labeled cell number (k) in hippocampal CA1 of IUE mice at P0. Scale bar = 100 μm. *n* = 12-15 slices/4 mice per group. Data are mean ± SEM. ^*^ *P* < 0.05, ^**^ *P* < 0.01, ^***^ *P* < 0.001 (in b, d P0, d P14, d P40, e P0, e P14, g, h, i, and k, *t*-test; in d E18, e E18, and e P40, Mann-Whitney Rank Sum Test).

**Supplementary Figure 2:**


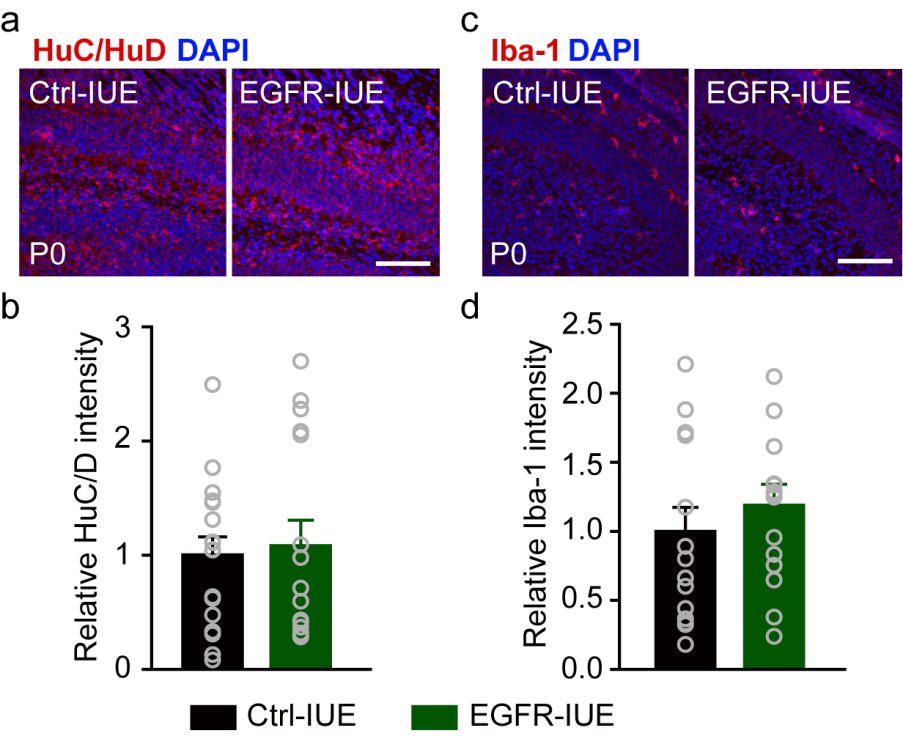


**Supplementary Figure 2 EGFR overexpression in developing astrocytes does not alter neurogenesis and microglia activation in P0 EGFR-IUE mice**. Related to FIGURE 1. (**a, b**) Representative images of immunohistostaining (a) and quantification of relative intensity (b) of HuC/HuD in hippocampal CA1 of IUE mice at P0. Scale bar = 100 μm. *n* = 16-17 slices/4 mice per group. (**c, d**) Representative images of immunohistostaining (c) and quantification of relative intensity (d) of Iba-1 in hippocampal CA1 of IUE mice at P0. Scale bar = 100 μm. *n* = 14-15 slices/4 mice per group. Data are mean ± SEM. Mann-Whitney Rank Sum Test in b and *t*-test in d.

**Supplementary Figure 3:**

**
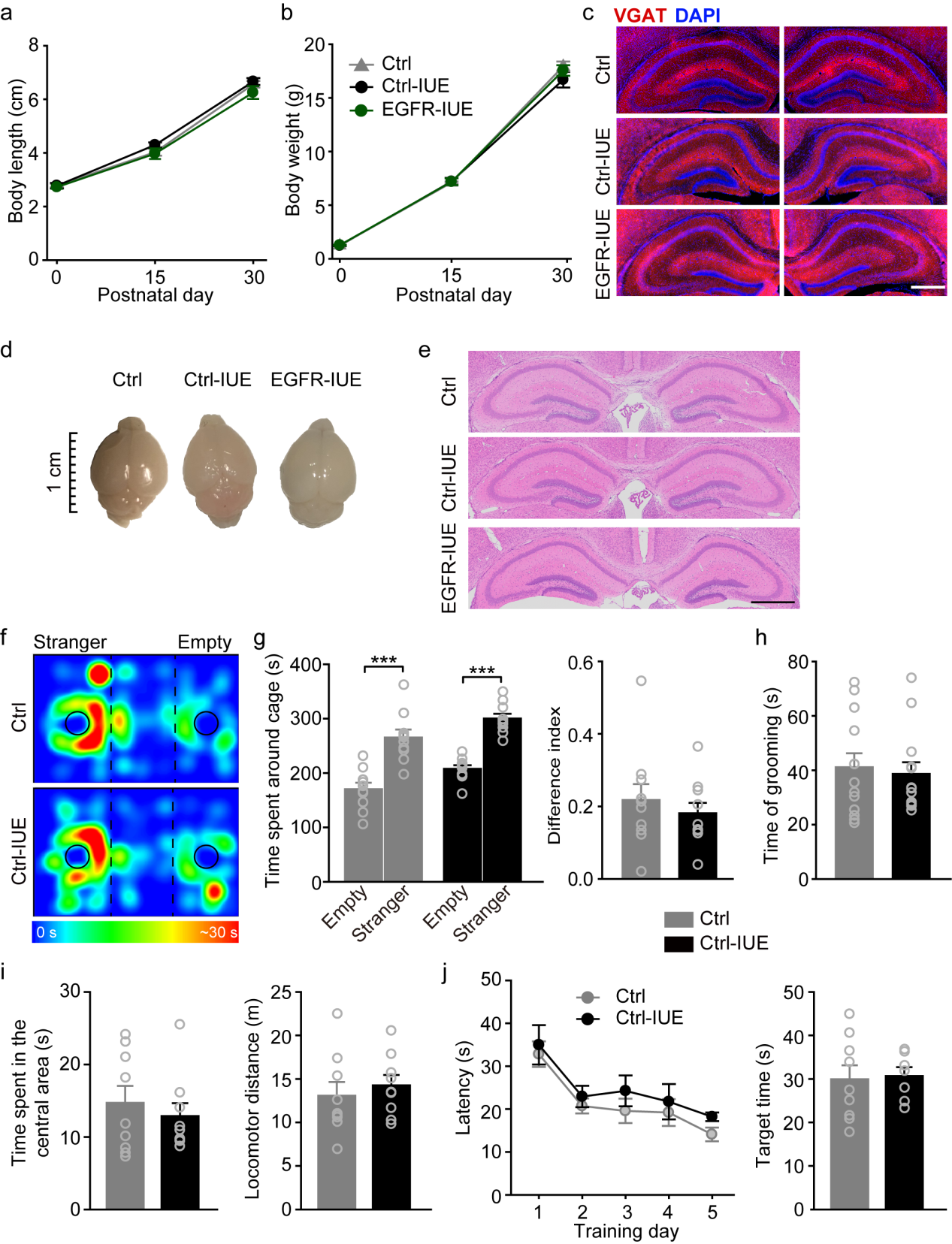
**

**Supplementary Figure 3 IUE does not affect general development or the relative behaviors in mice**. Related to FIGURE 1. (**a, b**) The average body length (a) and body weight (b) of the mice from Ctrl, Ctrl-IUE, and EGFR-IUE groups at P0, P15, and P30. *n* = 7-11 per group. (**c**) Representative sections of the hippocampi from P14 Ctrl, Ctrl-IUE, and EGFR-IUE mice stained with VGAT. Scale bar = 500 μm. (**d**) Photograph of brains from Ctrl, Ctrl-IUE, and EGFR-IUE mice at P14. (**e**) Hematoxylin and eosin (H&E) staining images of hippocampal sections from Ctrl, Ctrl-IUE, and EGFR-IUE mice at P14. Scale bar = 500 μm. (**f, g**) Representative heat maps of animal positions (f) and quantification of time spent in close proximity to the cages (g, left) and the difference index (g, right) in the three-chamber social interaction test. *n* = 10 per group. (**h**) Quantification of time spent grooming in Ctrl and Ctrl-IUE mice. *n* = 13 per group. (**i**) Quantification of time spent in the central area (left) and distances travelled (right) in the open field test. *n* = 9 per group. (**j**) Quantification of escape latency in each session of the hidden-platform test (left) and target quadrant searching time in the probe test (right) in the MWM task. *n* = 7-9 per group. Data are mean ± SEM. ^***^ *P* < 0.001 (in a, b, and j left, two-way ANOVA with post hoc Holm-Sidak test; in g left, one-way ANOVA with post hoc Bonferroni t-test; in g right, i, and j right, *t*-test; in h, Mann-Whitney Rank Sum Test).

**Supplementary Figure 4:**

**
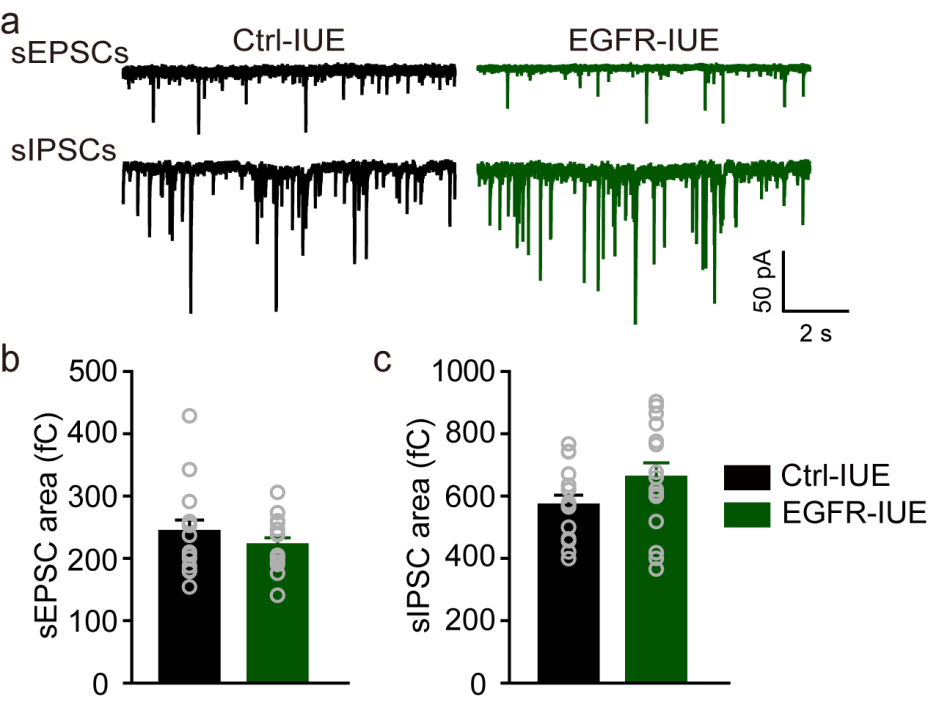
**

**Supplementary Figure 4 sEPSC and sIPSC responses recorded in the CA1 pyramidal neurons under pharmacological isolation in Ctrl-IUE and EGFR-IUE mice.** Related to FIGURE 2. (**a**) Representative traces of sEPSCs (top) and sIPSCs (bottom) both recorded at −70 mV in CA1 pyramidal neurons of Ctrl-IUE (black) and EGFR-IUE (green) mice. (**b, c**) Quantification of sEPSC (b) and sIPSC (c) charge transfers in Ctrl-IUE and EGFR-IUE mice. *n* = 15 slices/3 mice per group. Data are mean ± SEM. *t*-test.

**Supplementary Figure 5:**

**
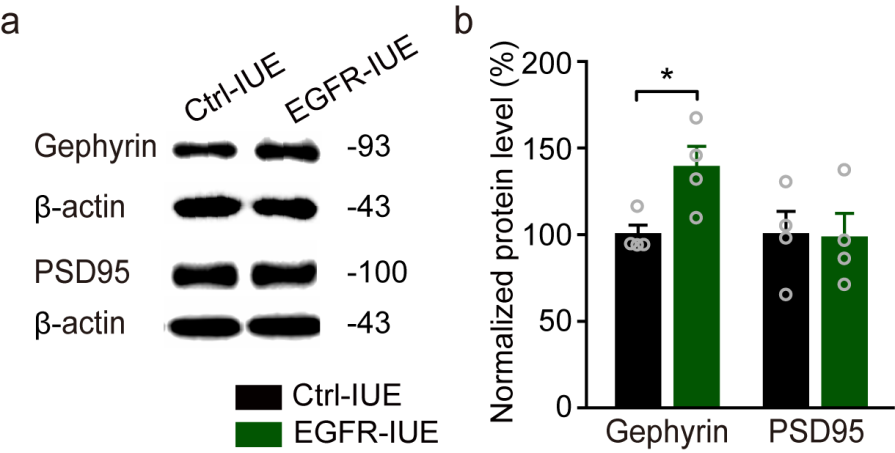
**

**Supplementary Figure 5 Increasing astrogenesis during development in the hippocampus selectively increases total protein level of gephyrin in CA1 area of the hippocampus**. Related to FIGURE 3. **(a, b**) Representative Western blots (a) and corresponding quantification of the total protein levels (b) of gephyrin and PSD95 in hippocampal CA1 of Ctrl-IUE and EGFR-IUE mice at P14. *n* = 4 per group. Data are mean ± SEM. ^*^ *P* < 0.05 (*t*-test).

**Supplementary Figure 6:**

**
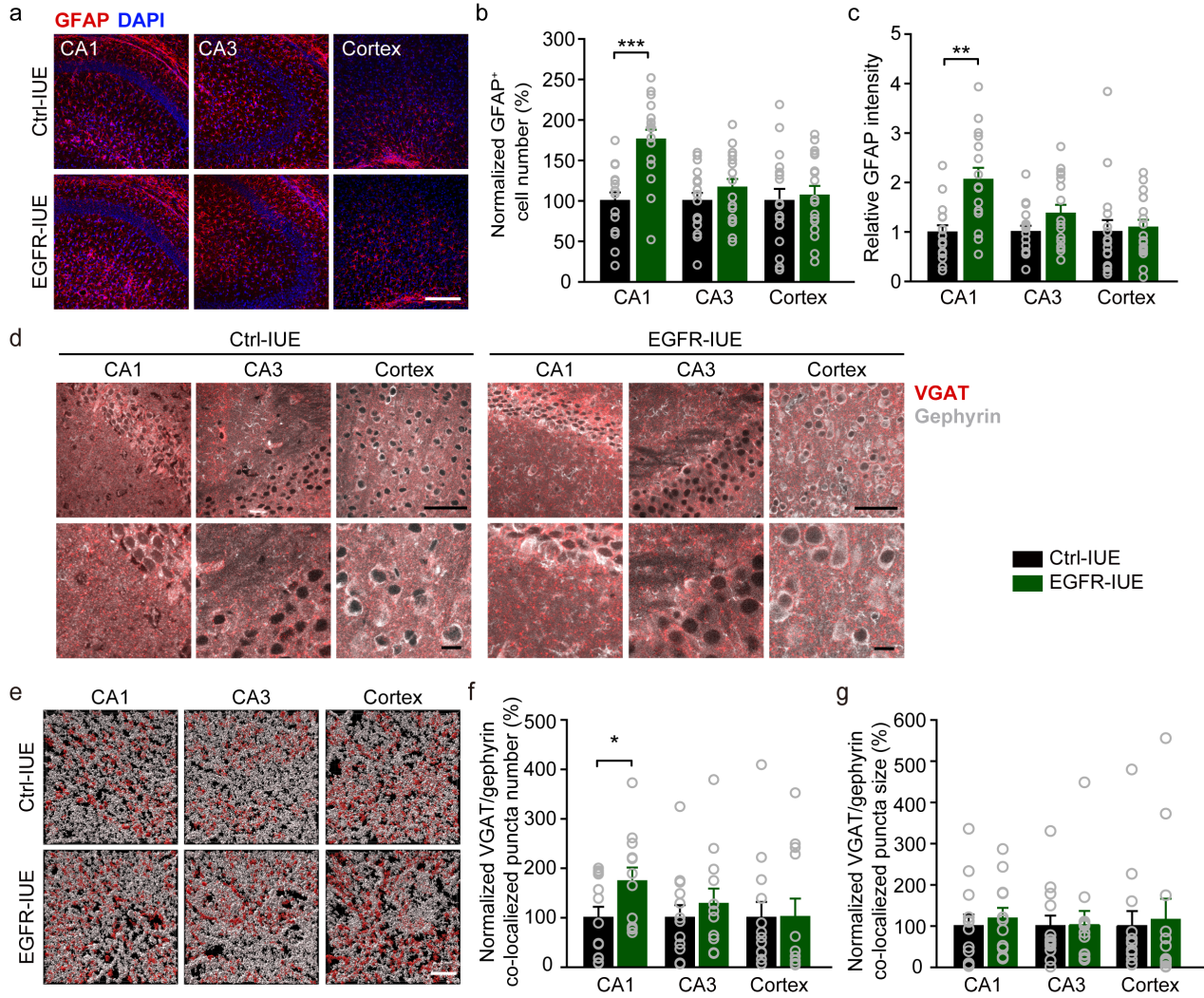
**

**Supplementary Figure 6 Increased inhibitory synapse formation is localized to the targeted CA1 area with high level of astrogenesis in the developing brain.** Related to FIGURE 3. (**a-c**) Representative images of immunohistostaining of GFAP (a) and quantification of normalized GFAP-positive (GFAP^+^) cell number (b) and relative GFAP intensity (c) in hippocampal CA1 and CA3 as well as cortical layer VI (Cortex) of Ctrl-IUE and EGFR-IUE mice. Scale bar = 100 μm. *n* = 16-17 slices/4 mice per group. (**d, e**) Representative images (d) and reconstructions (e) of VGAT/gephyrin co-localized puncta in hippocampal CA1 and CA3 as well as cortical layer VI of Ctrl-IUE and EGFR-IUE mice. Scale bars, 50 μm and 10 μm (d), 3 μm (e). (**f, g**) Quantification of the number (f) and size (g) of VGAT/gephyrin co-localized puncta in hippocampal CA1 and CA3 as well as cortical layer VI of Ctrl-IUE and EGFR-IUE mice. *n* = 12-13 slices/4 mice per group. Data are mean ± SEM. ^*^ *P* < 0.05, ^**^ *P* < 0.01, ^***^ *P* < 0.001 (in b CA1, c CA1, c cortex, f cortex, g CA3, and g cortex, Mann-Whitney Rank Sum Test; in b CA3, b cortex, c CA3, f CA1, f CA3, and g CA1, *t*-test).

**Supplementary Figure 7:**

**
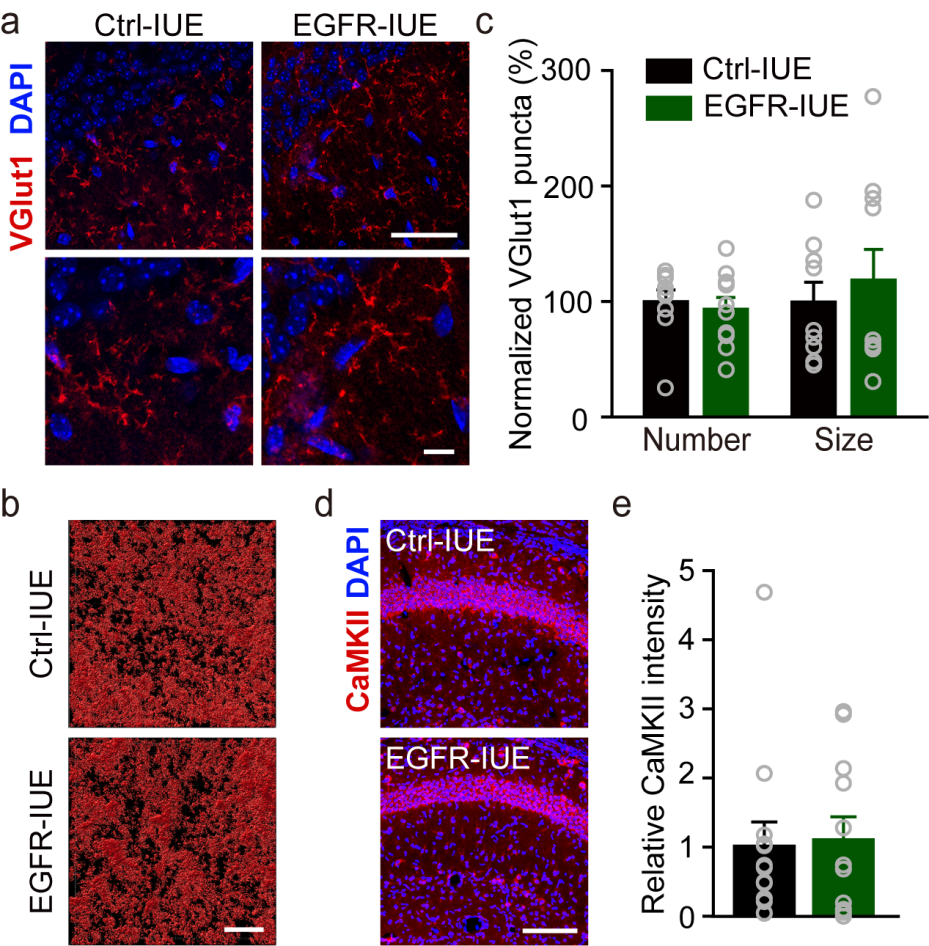
**

**Supplementary Figure 7 Increasing astrogenesis during development in the hippocampus does not affect excitatory synapse formation in CA1 area of the hippocampus**. Related to FIGURE 3. (**a, b**) Representative images (a) and reconstructions (b) of immunohistostaining of VGlut1 puncta in hippocampal CA1 of Ctrl-IUE and EGFR-IUE mice at P14. Scale bars, 50 μm and 10 μm (a), 3 μm (b). (**c**) Quantification of the number and size of VGlut1 puncta in hippocampal CA1 of Ctrl-IUE and EGFR-IUE mice. *n* = 9-10 slices/4 mice per group. (**d, e**) Representative images of immunohistostaining (d) and quantification of relative intensity (e) of CaMKII in hippocampal CA1 [stratum radiatum (SR)] of Ctrl-IUE and EGFR-IUE mice at P14. Scale bar, 100 μm. *n* = 12-13 slices/4 mice per group. Data are mean ± SEM. ^*^ *P* < 0.05 (in c number, *t*-test; in c size and e, Mann-Whitney Rank Sum Test).

**Supplementary Figure 8:**


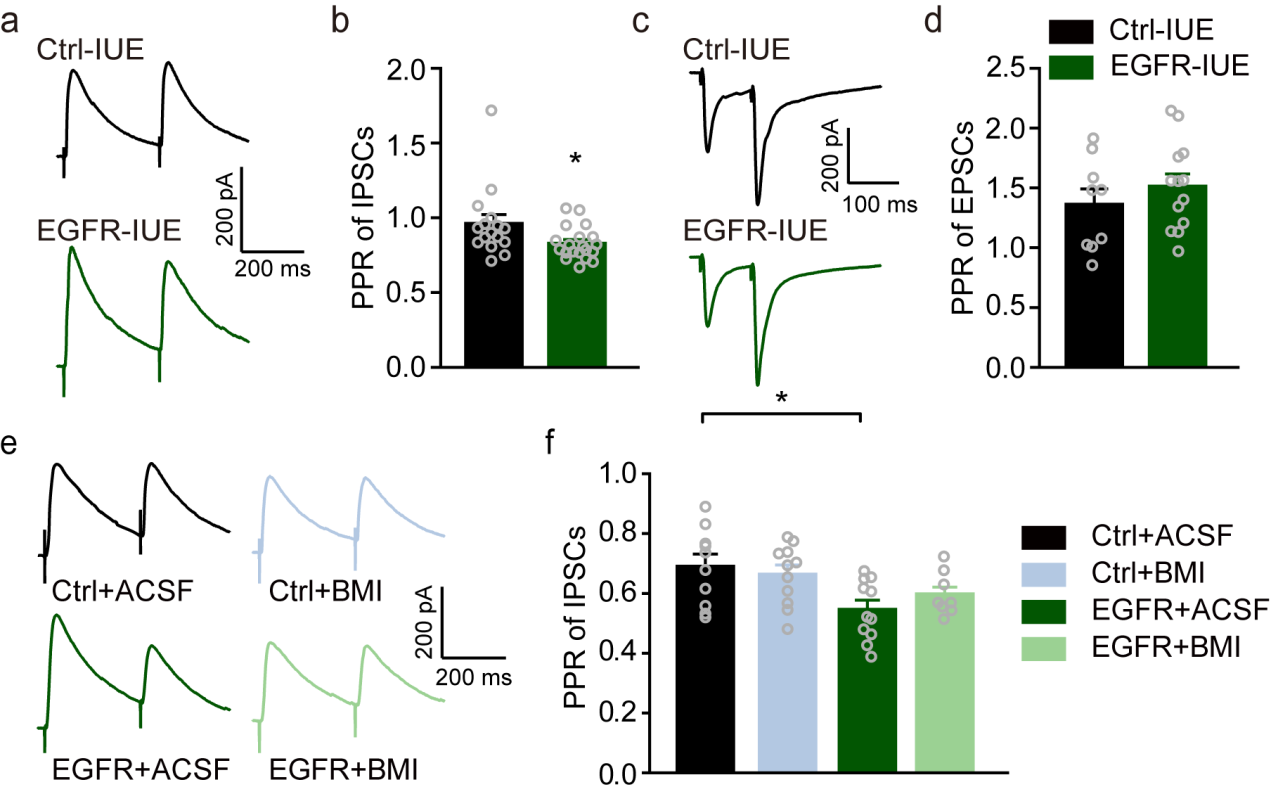


**Supplementary Figure 8 Increasing astrogenesis during development in the hippocampus causes a rise in presynaptic GABA release probability.** Related to FIGURES 3 and 5. (**a-d**) Representative traces of paired-pulse responses of evoked IPSCs (a) and EPSCs (c) at 40 μA and quantification of paired-pulse ratios (PPR) of evoked IPSCs (b) and EPSCs (d) in Ctrl-IUE and EGFR-IUE mice. *n* = 16-20 slices/3 mice per group for (b) and 9-13 slices/3 mice per group for (d). (**e, f**) Representative traces of paired-pulse responses (e) and quantification of PPR (f) of evoked IPSCs at 40 μA in BMI- or ACSF-treated Ctrl-IUE (Ctrl-BMI or Ctrl-ACSF) and EGFR-IUE (EGFR-BMI or EGFR-ACSF) mice. *n* = 9-11 slices/3 mice per group. Data are mean ± SEM. ^*^ *P* < 0.05 (in b, Mann-Whitney Rank Sum Test; in d, *t*-test; in f, One-way ANOVA with post hoc Bonferroni t-test).

**Supplementary Figure 9:**

**
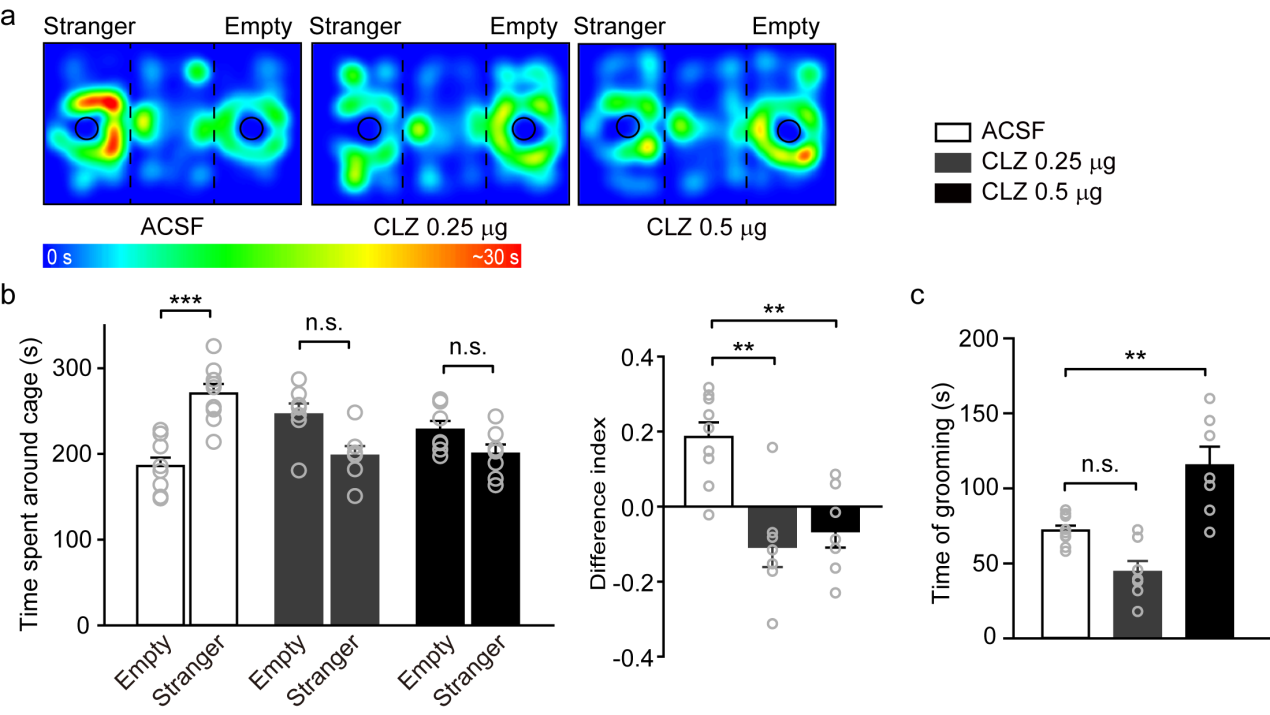
**

**Supplementary Figure 9 Localized increased GABAergic activity in CA1 area of the hippocampus by clonazepam (CLZ) is sufficient to induce an ASD-like behavioral phenotype in mice.** (**a**, **b**) Representative heat maps of animal positions (a) and quantification of time spent in close proximity to the cages (b, left) and the difference index (b, right) in the three-chamber social interaction test in vehicle mice received ACSF, 0.25 μg CLZ, or 0.5 μg CLZ treatments. *n* = 7-9 per group. (**c**) Quantification of time spent grooming in vehicle mice received ACSF, 0.25 μg CLZ, or 0.5 μg CLZ treatments. *n* = 7-9 per group. Data are mean ± SEM. ^**^*P* < 0.01, ^***^*P* < 0.001 (one-way ANOVA with post hoc Bonferroni t-test).

**Supplementary Figure 10:**

**
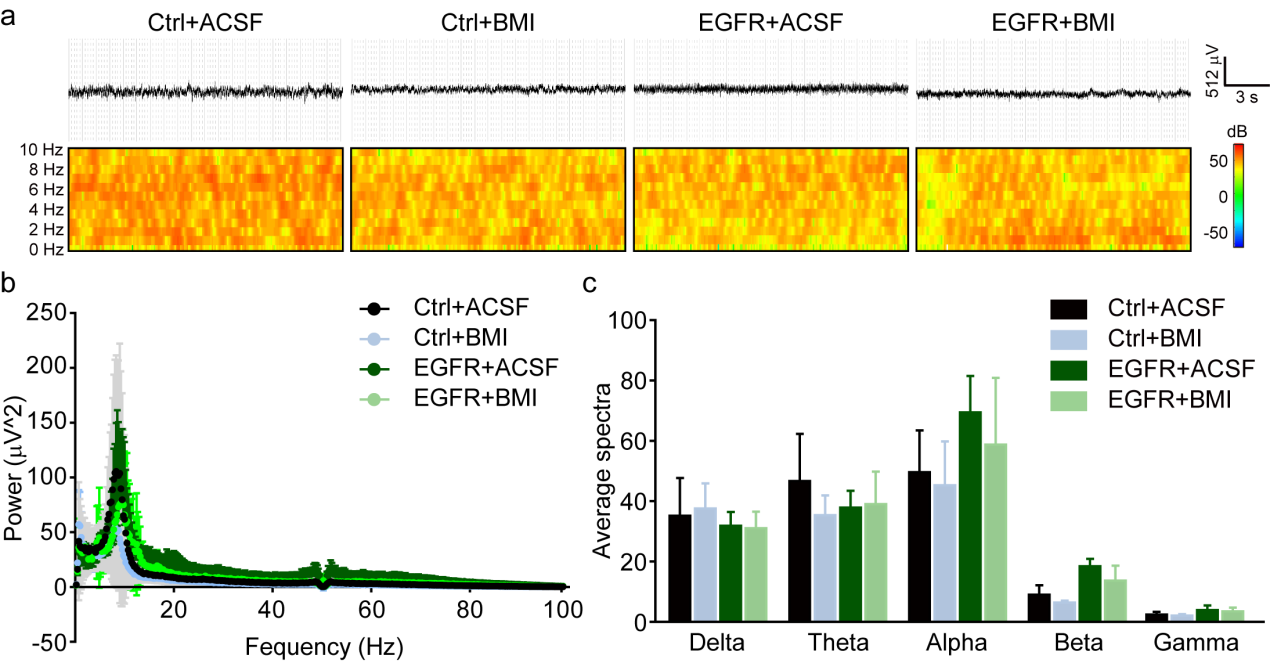
**

**Supplementary Figure 10 Intra-CA1 injection of BMI does not induce local seizure activity and affect excitatory network activity in the hippocampus of IUE mice.** Related to FIGURE 4. (**a**) Representative recording traces (top) and spectrograms (bottom) of EEGs recorded in the hippocampal CA1 of BMI- or ACSF-treated Ctrl-IUE (Ctrl-BMI or Ctrl-ACSF) and EGFR-IUE (EGFR-BMI or EGFR-ACSF) mice. (**b**) Corresponding EEG powers from BMI- or ACSF-treated Ctrl-IUE and EGFR-IUE mice. (**c**) The average spectra of different bands (Delta, Theta, Alpha, Beta, and Gamma) of EEG recordings from BMI- or ACSF-treated Ctrl-IUE and EGFR-IUE mice. *n* = 4 mice per group. Data are mean ± SEM. One-way ANOVA with post hoc Bonferroni t-test in b and two-way ANOVA with post hoc Holm-Sidak test in c.

**Supplementary Table 1** Average parameters of mIPSCs and mEPSCs recorded from IUE mice at P14. Related to FIGURE 2.

|  | mIPSCs | | mEPSCs | |
| --- | --- | --- | --- | --- |
| Parameters | Ctrl-IUE | EGFR-IUE | Ctrl-IUE | EGFR-IUE |
| Frequency, Hz | 2.29 ± 0.32 | 4.05 ± 0.44* | 0.46 ± 0.06 | 0.44 ± 0.04 |
| Amplitude, pA | 11.4 + 0.32 | 11.7 + 0.33 | 12.9 ± 1.53 | 11.9 ± 1.15 |
| Rise time, ms | 0.96 ± 0.06 | 1.05 ± 0.16 | 1.69 ± 0.18 | 1.81 ± 0.09 |
| Decay time, ms | 8.45 ± 1.29 | 8.11 ± 1.13 | 6.93 ± 0.39 | 8.01 ± 0.75 |
| Average area, fC | 571.8 ± 32.1 | 522.6 ± 17.6 | 292.1 ± 19.6 | 274.3 ± 16.1 |

*n* = 11-14 slices/3 mice per group. Data are mean ± SEM. *Indicates statistical significance between Ctrl-IUE and EGFR-IUE groups by *t*-test (or Mann-Whitney Rank Sum Test) with *P* < 0.05 or less.

**Supplementary Table 2** Excitatory and inhibitory synaptic responses recorded in CA1 pyramidal neurons from IUE mice at P40. Related to FIGURE 2.

| Parameters | Ctrl-IUE | EGFR-IUE |
| --- | --- | --- |
| mIPSC frequency, Hz | 2.91 ± 0.20 | 4.18 ± 0.42* |
| mIPSC amplitude, pA | 11.55 ± 0.36 | 11.75 ± 0.33 |
| mEPSC frequency, Hz | 0.63 ± 0.09 | 0.75 ± 0.08 |
| mEPSC amplitude, pA | 9.66 ± 0.56 | 9.01± 0.32 |
| Evoked IPSC amplitude, pA | 259.5 ± 24.6 | 413.0 ± 43.2* |
| Evoked IPSC PPR | 0.68 ± 0.05 | 0.53 ± 0.02* |
| sEPSC average area, fC | 285.4 ± 23.4 | 262.9 ± 18.0 |
| sIPSC average area, fC | 498.5 ± 18.0 | 586.0 ± 40.1 |
| E/I ratio | 0.36 ± 0.02 | 0.31 ± 0.01* |

*n* = 10-12 slices/3 mice per group. Data are mean ± SEM. *Indicates statistical significance between Ctrl-IUE and EGFR-IUE mice by *t*-test (or Mann-Whitney Rank Sum Test) or two-way ANOVA with *P* < 0.05 or less. The amplitudes of evoked IPSCs were quantified at stimulus intensity of 60 μA in both groups.

**Supplementary Table 3** Excitatory and inhibitory synaptic responses recorded in CA1 pyramidal neurons from Ctrl and Ctrl-IUE mice at P14. Related to FIGURE 2.

| Parameters | Ctrl | Ctrl-IUE |
| --- | --- | --- |
| mIPSC frequency, Hz | 2.02 ± 0.31 | 2.14 ± 0.25 |
| mIPSC amplitude, pA | 12.54 ± 1.15 | 12.34 ± 0.81 |
| mEPSC frequency, Hz | 0.54 ± 0.06 | 0.52 ± 0.06 |
| mEPSC amplitude, pA | 11.25 ± 0.68 | 11.55 ± 0.74 |
| sEPSC average area, fC | 344.0 ± 32.5 | 360.7 ± 41.0 |
| sIPSC average area, fC | 609.2 ± 53.7 | 597.1 ± 69.1 |
| E/I ratio | 0.36 ± 0.03 | 0.38 ± 0.03 |

*n* = 9 slices/3 mice per group. Data are mean ± SEM. Statistical significance between Ctrl-IUE and EGFR-IUE groups was analyzed by *t*-test (or Mann-Whitney Rank Sum Test).
